# Supplementary material for: Perspectives of the Friedreich ataxia community on gene therapy clinical trials
Source: Mol Ther Methods Clin Dev. 2023 Dec 18;32(1):101179. doi: 10.1016/j.omtm.2023.101179 (PMC10797190; doi:10.1016/j.omtm.2023.101179)
Supplement: Document S1. Survey materials and IRB approval letter [file mmc1.pdf]

OMTM, Volume *32*

Supplemental information

Perspectives of the Friedreich ataxia community on gene therapy clinical trials

Shandra J. Trantham, Mackenzi A. Coker, Samantha Norman, Emma Crowley, Julie Berthy, Barry J. Byrne, Sub Subramony, XiangYang Lou, and Manuela Corti

Email to Potential Participants:

Subject: Invitation to participate in FA Gene Therapy Survey

FARA is sharing information about a survey that is evaluating opinions about gene therapy. The results of this survey will help inform researchers, physicians, and biopharmaceutical companies about the FA community's knowledge and opinions on gene therapy. This information may also be utilized to guide future research and education for the FA community.

WHO CAN PARTICIPATE?

An individual living with FA or as a parent/guardian of a child living with FA and are a US resident.

- Individuals who complete the survey must be 14 years of age or older.
  - *Parents of children ages 14 to 17 years old, may elect to complete the survey together.*
- If the child is less than 14 years old, we ask that a parent complete the survey for them.

WHAT'S INVOLVED?

We ask you to read information about the survey, provide online consent, view a PowerPoint that has introductory information and then complete of an online survey. The online survey includes questions in regards to demographic and FA symptom and asks about your preferences and opinions related to gene therapy. The survey will take approximately 20 minutes to complete and all responses will be strictly confidential. The survey answers will be completely anonymous. Participants can skip any questions they do not wish to answer.

If you wish to participate in this survey, please click this link to participate:
https://redcap.ctsi.ufl.edu/redcap/surveys/?s=8L8XLYDXNJ

MORE INFORMATION:

If you have any questions or comments about this survey, please contact Mackenzi Coker. You can reach her at [REDACTED] or [REDACTED].

Thank you for considering participating in this important study.

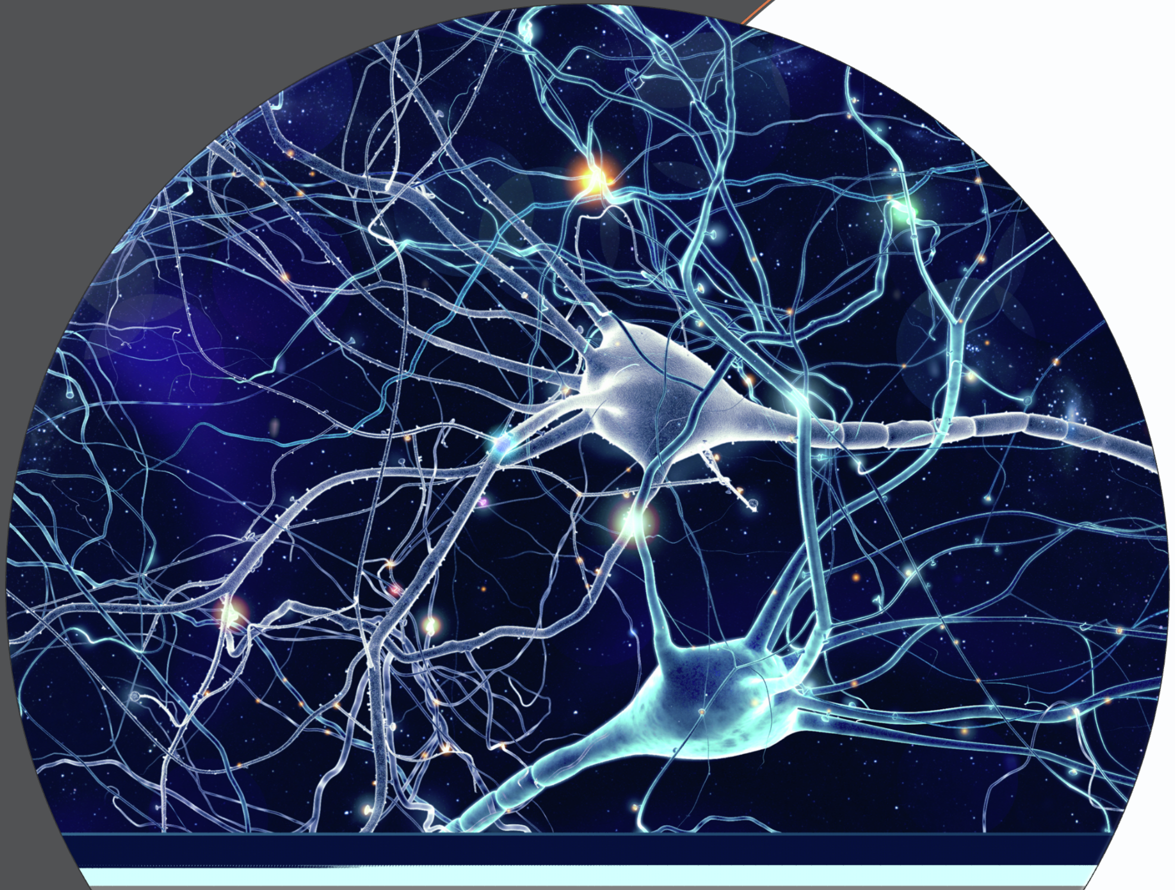

This study is a collaboration between UF and AavantiBio

Friedreich's Ataxia Gene Therapy Questionnaire

Introduction

UF | UNIVERSITY of FLORIDA

AAVANTIBIO

Introduction

Thank you for choosing to participate in this survey. This survey is being distributed through patient advocacy groups for Friedreich's ataxia (FA). The purpose is to collect opinions about gene therapy research for FA. Patients with FA or their family members may take the survey. The results will be used to help develop future clinical studies for FA.

If you are 14 years old or younger, please ask a parent or caregiver to complete the survey for you. Before answering the questions, please review this PowerPoint and read the additional information from the link.

UF UNIVERSITY of FLORIDA AAVANTIBIO

FA and Gene Therapy

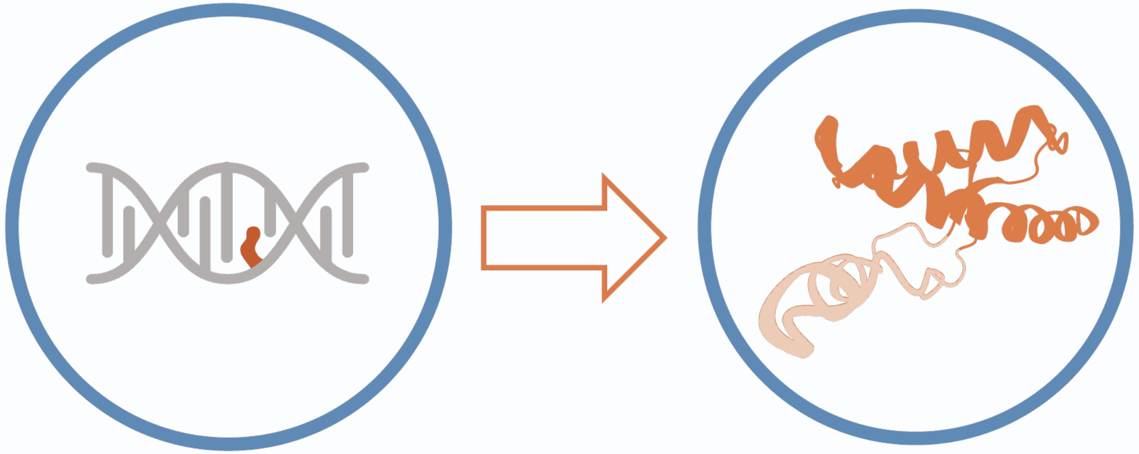

Mutated *FXN* gene

Missing or defective frataxin protein

FA is a rare disease that is caused by defects (mutations) in the gene for a protein called frataxin. These mutations interfere with the production or function of frataxin. Lack of frataxin leads to the symptoms of FA.

UF UNIVERSITY of FLORIDA

AAVANTIBIO

FA and Gene Therapy

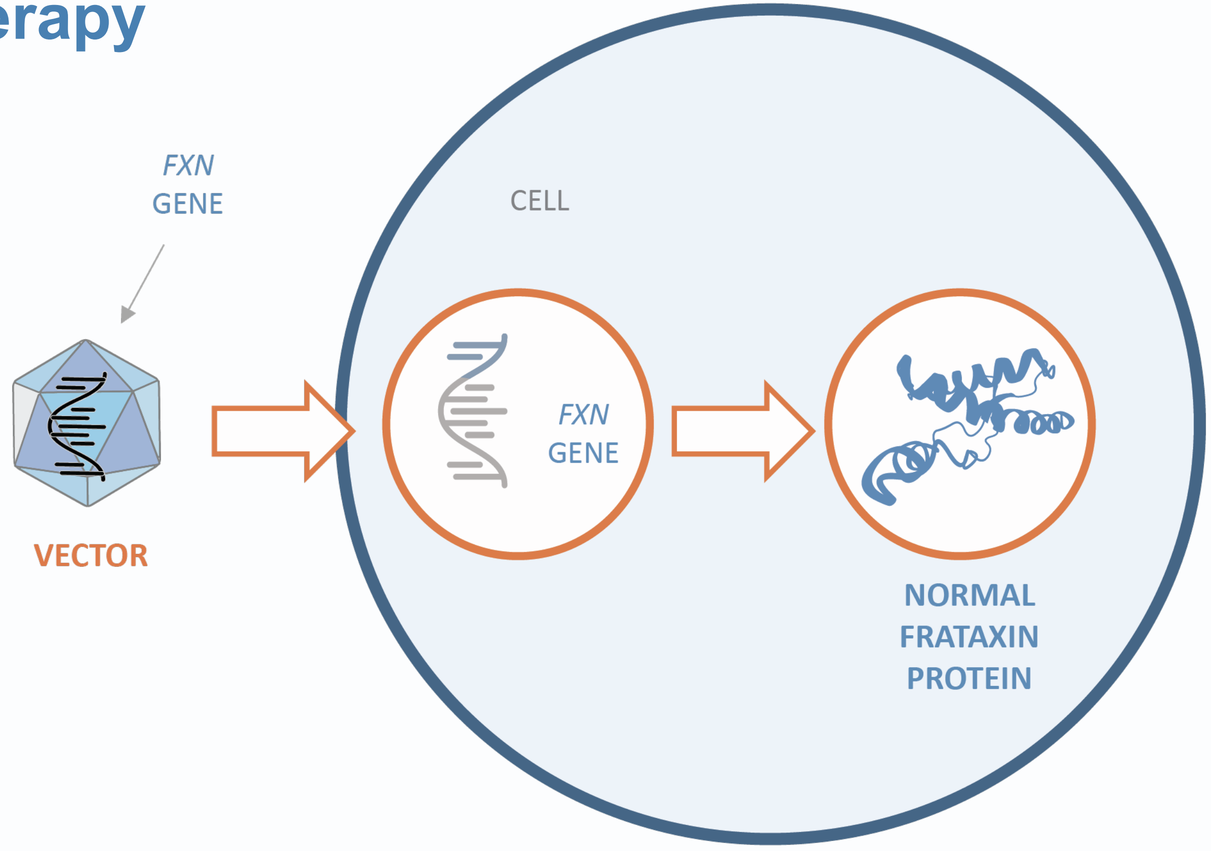

Gene therapy is a potential treatment for FA. Gene therapy works by putting new genes for frataxin into cells to restore function. The genes provide instructions for making frataxin protein. Increasing the amount of frataxin in cells may help reduce the severity of FA. These genes are delivered by a vehicle called a vector. The vector is a modified virus that does not cause any disease.

UF UNIVERSITY of FLORIDA

AAVANTIBIO

FA and Gene Therapy

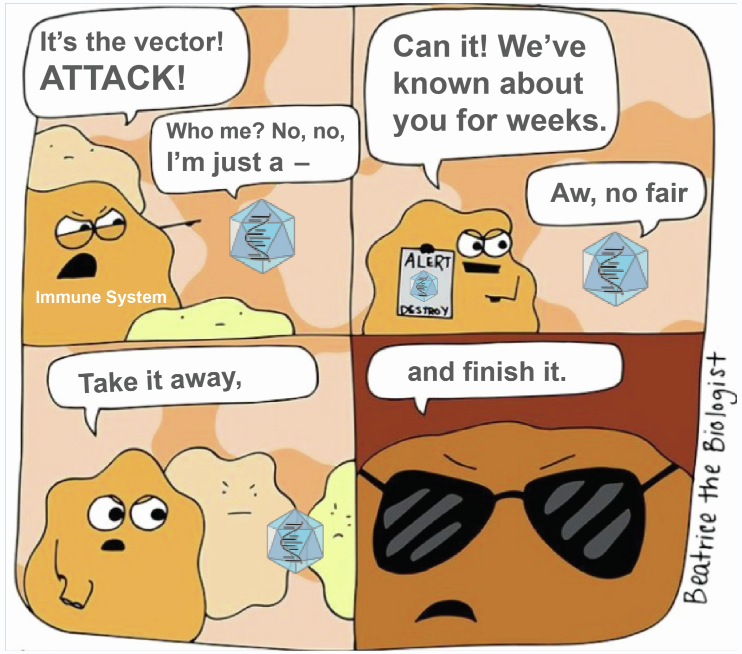

Currently, patients who receive gene therapy can only be treated once. This is because the body's defense system (immune system) remembers the vector. If it sees the vector again, it will attack the vector and prevent it from working.

Researchers are trying to find ways to prevent this from happening. If they are successful, patients may be able to receive gene therapy more than once. This may help gene therapy last longer. It could also allow patients to participate in future studies (clinical trials) on gene therapy.

Figure adapted from McKissick K. Get Your Flu Shot. Beatrice the Biologist. November 7, 2014. Accessed November 9, 2021. www.beatricebiologist.com/2014/

UF UNIVERSITY of FLORIDA AAVANTIBIO

FA and Gene Therapy

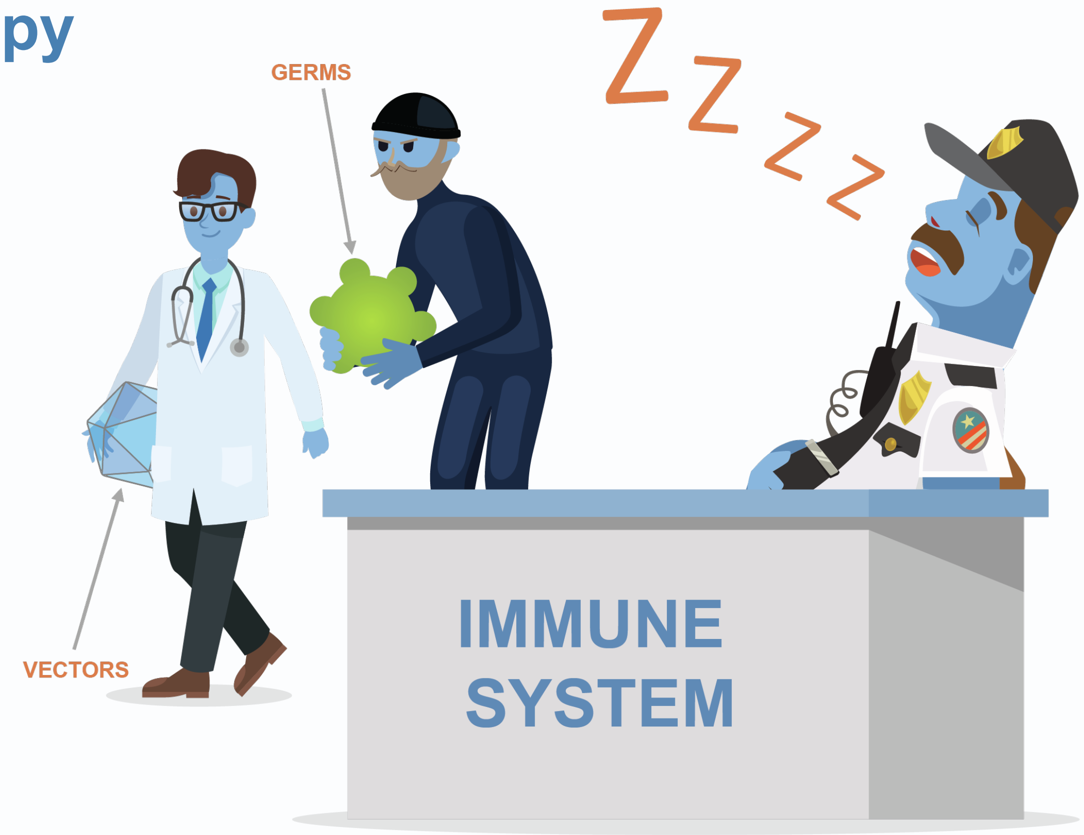

Researchers are testing medicines that change how the immune system works. This is called immunomodulation. Immunomodulation medicines could be used with gene therapy. Then the immune system would not attack the vector. However, immunomodulation medicines may make common illnesses, such as a cold or flu, more severe than usual. This is why doctors and nurses closely monitor patients who are treated with these medicines.

Figure adapted from media.istockphoto.com/vectors/guardpoliceman-napping-vector-id120881647

UF UNIVERSITY of FLORIDA AAVANTIBIO

Gene Therapy and Clinical Trials

Placebo-Controlled Clinical Trial

Researchers perform clinical trials to test whether medicines can improve people’s health.

There are different kinds of clinical trials. One is called a placebo-controlled study. Participants are divided into 2 groups. One group is given an inactive, harmless treatment (placebo). The other group is treated with the new medicine.

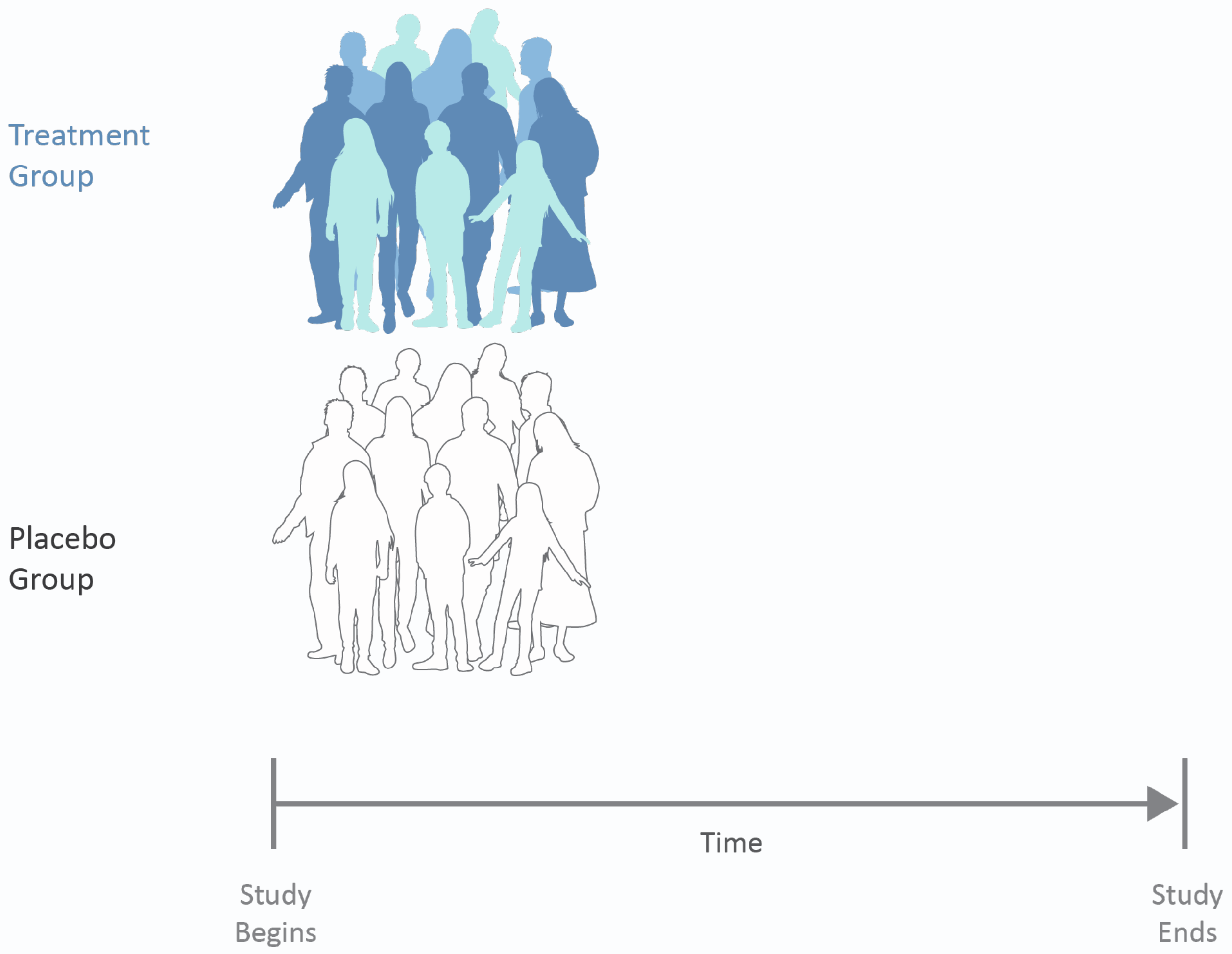

UF UNIVERSITY of FLORIDA

AAVANTIBIO

Gene Therapy and Clinical Trials

Delayed-Treatment Clinical Trial

Because of the potential benefit, trial designs try to minimize size and length of placebo. In some cases , researchers can perform a delayed-treatment clinical trial. Participants are divided into 2 groups. One group receives the potential new medicine immediately. The placebo group receives the same active treatment after a period of time.

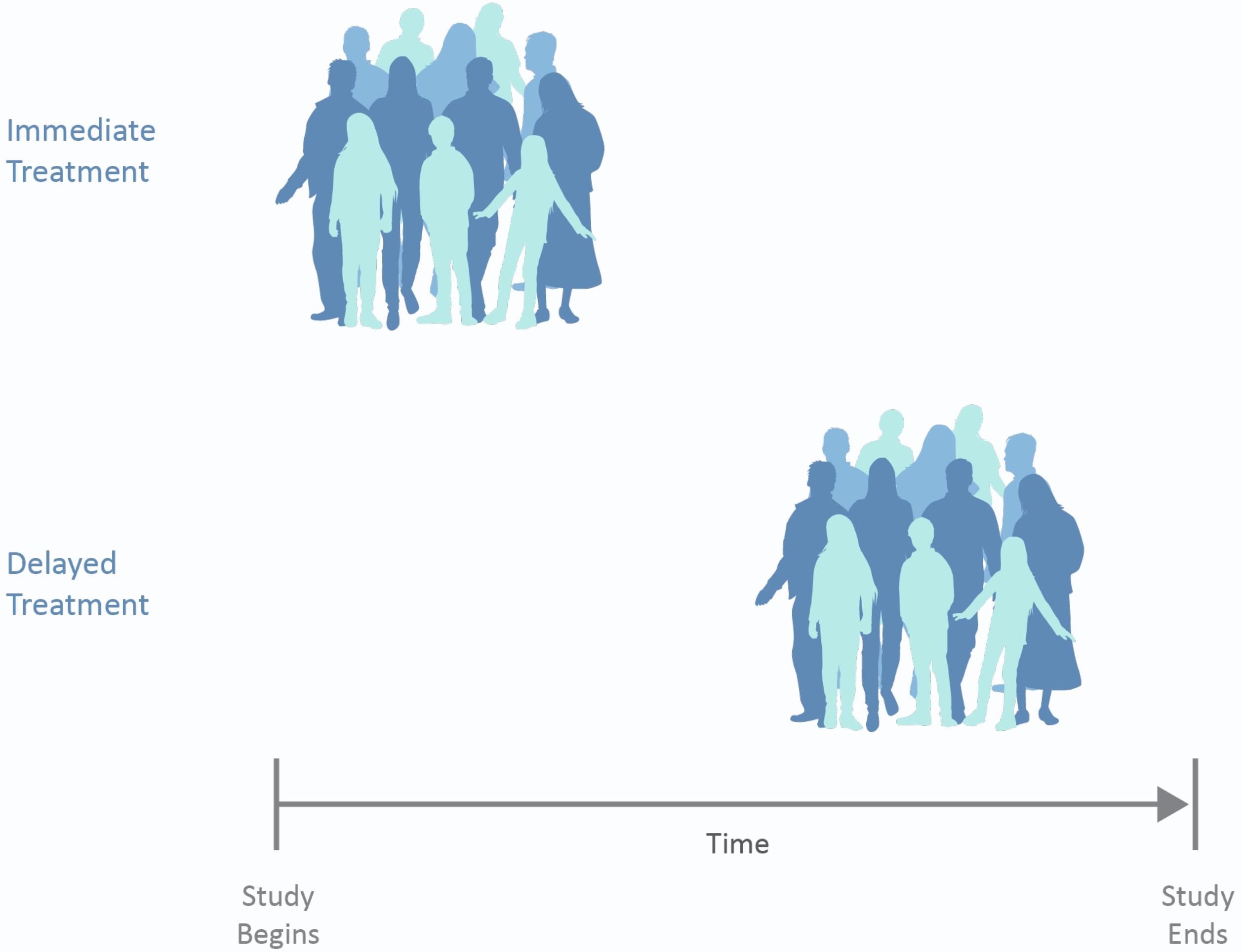

UF UNIVERSITY of FLORIDA AAVANTIBIO

Back to RedCap

Now that you have reviewed the FA Questionnaire Introduction, please head back to the RedCap link to read the additional information and complete the survey, if you agree to participate.

UF | UNIVERSITY of FLORIDA   AAVANTIBIO

Title of Project: Friedreich's Ataxia Gene Therapy Questionnaire

Principal Investigator: Manuela Corti, Assistant Professor
Powell Center for Rare Disease Research and Therapy, University of Florida

1. **Purpose of the Study:** This survey collects opinions about gene therapy research for Friedreich's ataxia (FA). Patients with FA or their family members may take the survey.
2. **Instructions to be Followed:** You will be asked to read a 1-page introduction and answer 42 questions.
3. **Duration:** The survey will take about 15 minutes.
4. **Benefits of Participating:** There is no direct benefit to you. The survey results may benefit others.
5. **Risks of Participating:** There is minimal risk.
6. **Confidentiality:** Your participation in this survey is confidential. Your responses are not linked to your name. If the survey results are published, researchers will not identify you.
7. **Right to Ask Questions:** Please call Mackenzi Coker at 352-294-8754 if you have any questions or concerns about this study.
8. **Privacy Authorization**: University of Florida and Shands Hospital can only share your medical information with the following people:
   a. Other researchers conducting approved research
   b. The sponsor of a research study
   c. United States government agencies
9. **Voluntary Participation:** Your decision to participate in this survey is voluntary. You can stop at any time. You may skip any question that you do not want to answer. If you choose not to participate, this will not affect your current or future healthcare at Shands Hospital or University of Florida. If you have any questions about your rights as a participant, please call the Institutional Review Board at 352-273-9600.

Completion of the survey means that you have read the information above. It also means that you agree to take part in the study. Please keep this information for your records.

CONFIDENTIAL

Thank you for choosing to participate in this survey. This survey is being distributed through patient advocacy groups for Friedreich's ataxia (FA). The purpose is to collect opinions about gene therapy research for FA. Patients with FA or their family members may take the survey. The results will be used to help develop future clinical studies for FA.

If you are 14 years old or younger, please have a parent or caregiver complete the survey for you. Before answering the questions, please download the Introductory PowerPoint and then read the information below.

Please ensure you have reviewed the Introductory PowerPoint, prior to reading the information below.

FA and Gene Therapy

FA is a rare disease that is caused by defects (mutations) in the gene for a protein called frataxin. These mutations interfere with the production or function of frataxin. Lack of frataxin leads to the symptoms of FA.

Gene therapy is a potential treatment for FA. Gene therapy works by inserting new genes for frataxin into cells to restore function. The genes provide instructions for making frataxin protein. Increasing the amount of frataxin in cells may help reduce the severity of FA. These genes are delivered by a vehicle called a vector. The vector is a modified virus that does not cause any disease.

Currently, patients who receive gene therapy can only be treated once. This is because the body's defense system (immune system) remembers the vector. If it sees the vector again, it will attack the vector and prevent it from working. Researchers are trying to find ways to prevent this from happening. If they are successful, patients may be able to receive gene therapy more than once. This may help gene therapy last longer. It could also allow patients to participate in future studies (clinical trials) on gene therapy.

In addition to the body potentially attacking the vector, there are other potential side effects / risks that have been observed in human gene therapy trials. Some of those side effects include, but are not limited to: Nausea and vomiting, low platelet counts, fever, allergic reactions, liver inflammation, tissue injury, headache, generalized inflammation, cardiac problems, and abnormal blood tests. Please keep in mind that gene therapy research remains relatively new, therefore, all side effects / risks are not known. In (very) rare cases side effects can be severe and lead to death. Researchers are testing medicines that change how the immune system works. This is called immunomodulation. Immunomodulation medicines could be used with gene therapy. Then the immune system would not attack the vector, and could help reduce the risk / alleviate some of the known side effects. However, immunomodulation medicines may make common illnesses, such as a cold or flu, more severe than usual. This is why doctors and nurses closely monitor patients who are treated with these medicines.

Gene Therapy and Clinical Trials

It is anticipated that gene therapy will improve a patient's condition. When tested in other conditions the patient's condition has greatly improved.

Researchers perform clinical trials to test whether medicines are safe and can improve people's health. There are different kinds of clinical trials. One is called a placebo-controlled study. Participants are divided into 2 groups. One group is given an inactive, harmless treatment (placebo). The other group is treated with the potential new medicine.

Because of the potential benefit, trial designs try to minimize size and length of placebo. In some cases, researchers can perform a delayed-treatment clinical trial. Participants are divided into 2 groups. One group receives the potential new medicine immediately. The placebo group receives the same active treatment after a period of time. However some participants in the placebo group may become ineligible by the time they are able to receive the medicine.

Some clinical trials have more than 1 treatment group. Researchers may compare different amounts (doses) of the potential new medicine. This is called a dose-escalation study. In a dose-escalation study, a lower dose is studied

CONFIDENTIAL

first. If the lower dose is safe, a higher dose may be tested next. This type of clinical trial helps researchers determine which dose works best and is safest. A higher dose may be more effective but may have more side effects.

Consent to Participate in This Study

Thank you for choosing to participate in this survey. If you are 14 years old or younger, please ask a parent or caregiver to complete the survey for you. Your participation is voluntary. You do not have to answer any questions that you do not want to. You may stop the survey at any time. All answers will be kept anonymous. There is minimal risk with your participation. Please indicate below if you are willing to participate.

☐ I have read and understand the information above to the best of my ability. I am aware that my participation is voluntary. I understand that my answers are not linked to my name.

If you have any questions or comments about this survey, please contact Mackenzi Coker. You can reach her at mcoker@peds.ufl.edu or 352-294-8754.

Please continue to the next page to begin the survey.

Please answer the questions below as accurately as possible.

1) Who is completing this survey? (Choose one)

Parent/Caregiver of patient with Friedreich's ataxia (FA) ☐ Patient with FA ☐

2) What is your/the patient's current age? ___________

3) What is your/the patient's gender?

Male ☐ Female ☐ Non-Binary ☐ Prefer not to answer ☐

4) How old were you/was the patient at the time of diagnosis? __________

5) How old were you/was the patient when symptoms were first noticed? __________

6) What was the first symptom you/the patient experienced? This includes symptoms experienced prior to diagnosis. (Choose one)

Balance/Inability to Walk	☐
Impaired Coordination	☐
Fidgetiness	☐
Fatigue	☐
Scoliosis (abnormal curvature of the spine)	☐
Cardiomyopathy or Heart Problems	☐
Speech, Vision, or Hearing Problems	☐
Other (Please specify:)	☐

7) Which of the following symptoms most interferes with your/the patient's quality of life today? (Choose one) (Choose one)

Balance/Inability to Walk	☐
Impaired Coordination	☐

CONFIDENTIAL

Fidgetiness ☐
Fatigue ☐
Scoliosis (abnormal curvature of the spine) ☐
Cardiomyopathy or Heart Problems ☐
Speech, Vision, or Hearing Problems ☐
Other (Please specify:) ☐

8) If a medicine could treat only one of your/the patient's symptoms, which would you/the patient prefer? (Choose one)

Balance/Inability to Walk ☐
Impaired Coordination ☐
Fidgetiness ☐
Fatigue ☐
Scoliosis (abnormal curvature of the spine) ☐
Cardiomyopathy or Heart Problems ☐
Speech, Vision, or Hearing Problems ☐
Other (Please specify:) ☐

9) Have you/Has the patient previously participated in any clinical trial?

Yes ☐ No ☐

10) Please share your/the patient's opinions about the following statements. (Choose one option in each row.)

	Strongly Disagree	Disagree	Neither Agree nor Disagree	Agree	Strongly Agree
10.1. I think clinical trials are important for scientific research.					
10.2. Clinical trials are necessary to study the effects of treatments for FA.					
10.3. I am aware of scientific advances in FA.					
10.4. My healthcare providers make me aware of advances in treatment for FA.					
10.5. My healthcare providers make me aware of upcoming clinical trials for FA.					
10.6. Patient advocacy organizations for FA make me aware of upcoming clinical trials.					
10.7. I learn about upcoming clinical trials through the internet.					
10.8. I am aware of at least 1 clinical trial for FA.					
10.9. Clinical trials for FA are focused on patient medical needs ~~needs~~.					

CONFIDENTIAL

10.10. Patient preferences ~~opinions~~ are incorporated into clinical trials for FA.					

11) Prior to this survey, had you/the patient heard about gene therapy?

Yes ☐ No ☐ I don’t know ☐

12) Please share your/the patient’s opinions about the following statements. (Choose one option in each row.)

	Strongly Disagree	Disagree	Neither Agree nor Disagree	Agree	Strongly Agree
12.1. Prior to this survey, I was knowledgeable about gene therapy.					
12.2. Prior to this survey, I was knowledgeable about different types of clinical trials.					
12.3. My knowledge of gene therapy improved after reading the survey introduction.					
12.4. Gene therapy will lessen my symptoms.					
12.5. Gene therapy will prevent my symptoms from worsening.					
12.6. Gene therapy will cure me of FA.					
12.7. I would be more likely to join a clinical trial if researchers consider my preferences about the study design.					
12.8. I would be comfortable sharing my preferences about clinical trial study design.					
12.9. I understand that gene therapy has risks.					

CONFIDENTIAL

13) Please share your/the patient's opinions about joining a gene therapy clinical trial. (Choose one option in each row.)

	Strongly Disagree	Disagree	Neither Agree nor Disagree	Agree	Strongly Agree
13.1. I would join a gene therapy study, even if it means I could not receive gene therapy again in the future.					
13.2. I would join a gene therapy study regardless of the consequences and possible side effects.					
13.3. I believe the most severe patients with FA should be treated first.					
13.4. I believe mildly affected patients with FA should be treated first.					
13.5. Children with FA should be treated with gene therapy before adult patients.					
13.6. If I joined a gene therapy study, I would prefer **not** to be treated with immunomodulation medicines.					
13.7. I would join a gene therapy study regardless of whether I was treated with immunomodulation medicines.					
13.8. If I joined a gene therapy study, the vector dose would not matter to me.					
13.9. If I joined a gene therapy study, I would prefer to be treated immediately, even if I receive a lower dose.					
13.10. If I joined a gene therapy study, I would prefer to be treated later if I could receive a higher dose.					
13.11. I believe that all patients in a clinical study should be treated with the same dose of a medication to ensure fairness.					
13.12. The time commitment would influence my decision to participate.					
13.13. My decision to participate would be influenced based on available and upcoming trials.					

CONFIDENTIAL

Institutional Review Board
UNIVERSITY of FLORIDA

Health Center Institutional Review Board
FWA00005790

PO Box 100173
Gainesville FL 32610-0173
Telephone: (352) 273−9600
Facsimile: (352) 273−9614
Email: irb@ufl.edu

DATE: 1/29/2020
TO: Manuela Corti
2004 Mowry Road - 2nd floor
Gainesville, Florida 32610
FROM: Peter Iafrate, IRB Chairman, University of Florida
Chair IRB-01
IRB#: **IRB201800502**
TITLE: Friedreich's Ataxia Gene Therapy Questionnaire

**Approved as Expedited**	**Expires on: 1/23/2023**

You have received IRB approval to conduct the above-listed research project. Approval of this project was granted on 1/23/2020 by IRB-01. This study is approved as expedited because it poses minimal risk and is approved under the following expedited category:

> 7. Research on individual or group characteristics or behavior (including, but not limited to, research on perception, cognition, motivation, identity, language, communication, cultural beliefs or practices and social behaviors) or research employing survey, interview, oral history, focus group, program evaluation, human factors evaluation or quality assurance methodologies. Note: Some research in this category may be exempt from the regulations for the protection of human subjects as noted in 45 CFR 46.101(b)(2) and (b)(3). This listing refers only to research that is not exempt.

Approval Includes, but is not limited to:

FA Questionnaire IRB Protocol

*****Email Script*****

Consent Waiver Type:

Waiver of Documentation of Informed Consent

The researcher will still inform the potential subject about the research and seek to obtain consent, sometimes by including an IRB approved written statement that includes the mandatory elements of consent. However, consent of the subject is not documented by having the subject sign an Informed Consent form.

HIPAA Waiver Type:

to enroll subjects in the study

Principal Investigator Responsibilities:

The PI is responsible for the conduct of the study. Please review these responsibilities described at: http://irb.ufl.edu/irb01/researcher-information/researcherresponsibilities.html
Important responsibilities described at the above link include:

- Using currently approved consent form to enroll subjects (if applicable)
- Renewing your study before expiration
- Obtaining approval for revisions before implementation
- Reporting Adverse Events
- Retention of Research Records
- Obtaining approval to conduct research at the VA
- Notifying other parties about this project's approval status

Study Team:

Emma	Crowley	Study Coordinator
Samantha	Norman	Study Coordinator

The Foundation for The Gator Nation
An Equal Opportunity Institution
Confidentiality Notice: This e-mail message, including any attachments, is for the sole use of the intended recipient(s), and may contain legally privileged or confidential information. Any other distribution, copying, or disclosure is strictly prohibited. If you are not the intended recipient, please notify the sender and destroy this message immediately. Unauthorized access to confidential information is subject to federal and state laws and could result in personal liability, fines, and imprisonment. Thank you.
