# Supplementary material for: Perspectives of the Friedreich ataxia community on gene therapy clinical trials
Source: Mol Ther Methods Clin Dev. 2023 Dec 18;32(1):101179. doi: 10.1016/j.omtm.2023.101179 (PMC10797190; doi:10.1016/j.omtm.2023.101179)
Supplement: Document S2. Article plus supplemental information [file mmc2.pdf]

Molecular Therapy
Methods & Clinical Development
Original Article

Perspectives of the Friedreich ataxia community on gene therapy clinical trials

Shandra J. Trantham,[1,2] Mackenzi A. Coker,[2] Samantha Norman,[2] Emma Crowley,[2] Julie Berthy,[2] Barry J. Byrne,[2] Sub Subramony,[3] XiangYang Lou,[4] and Manuela Corti[2]

[1]Genetics and Genomics Graduate Program, University of Florida, Gainesville, FL 32611, USA; [2]Department of Pediatrics, College of Medicine, University of Florida, Gainesville, FL 32611, USA; [3]Department of Neurology, College of Medicine, University of Florida, Gainesville, FL 32611, USA; [4]Department of Biostatistics, College of Public Health and Health Professions, University of Florida, Gainesville, FL 32611, USA

Gene therapy is a potential treatment for Friedreich ataxia, with multiple programs on the horizon. The purpose of this study was to collect opinions about gene therapy from individuals 14 years or older with Friedreich ataxia or parents/caregivers of Friedreich ataxia patients who were diagnosed as children 17 or younger. Participants were asked to complete a survey after reading brief educational materials regarding gene therapy. Most of the patients captured in this survey have an early-onset (classical) presentation of the disease. Participants expressed urgency in participating in gene therapy clinical trials despite the associated risks. About half of the respondents believed that gene therapy would cease progression or minimize symptoms, whereas nearly one-fourth expected to be cured. The survey also revealed how participants perceive their symptom burden, because a substantial majority reported that balance/walking issues most interfere with their quality of life and would be the symptom they would prioritize treating. Although not statistically significant, more caregivers prioritized treating cardiomyopathy than patients. This study provides valuable information on priorities, beliefs, and expectations regarding gene therapy and serves to guide future gene therapy opinion studies and gene therapy trial design.

INTRODUCTION

Friedreich ataxia (FA) is a rare, progressive, neuromuscular disease caused by a recessive mutation in the frataxin (*FXN*) gene that limits the production of frataxin protein.[1–3] Although FA is a rare disease, it is the most common hereditary ataxia, affecting an estimated 1 in 20,000 individuals of Western European ancestry.[4] The typical onset of disease is before age 15 years, and patients exhibit some combination of gait and limb ataxia, loss of coordination, fatigue, scoliosis, dysphagia, hearing loss, vision loss, saccadic eye movements, and cardiac involvement.[5,6] Patients typically require the use of a wheelchair within 5–10 years of diagnosis and eventually require assistance with all activities of daily living in later stages of the disease.[5,6] The cardiac disease associated with FA, cardiomyopathy, affects approximately 60% of patients and is typically fatal, putting the median age of death at 35 years.[7,8] Patients with an onset of disease after age 25 (late-onset FA) generally have a less severe course of progression, with an absence of scoliosis and cardiac involvement.[1] As of now, there is one recently US Food and Drug Administration (FDA)–approved treatment for FA, omaveloxolone (Skyclarys).[9] However, there are no FDA-approved treatments that target the root etiology: low levels of frataxin.[10] The lack of treatments targeting the root etiology presents a substantial unmet medical need.

There is interest in the development of a gene replacement therapy (referred as gene therapy hereafter) to provide a healthy copy of the FXN gene and in theory, restore frataxin protein to a nonpathogenic level. Several investigational gene therapies are currently in preclinical and clinical development for FA that differ in their desired treatment target, the area that a therapeutic is designed to reach. Different vectors and routes of administration can be used to target different areas of the body.[11] Since FA is a multisystem disease, the choice of treatment target presents a challenge for gene therapy. Different routes of administration may be required to treat cardiac and nervous system involvement. The main sites of neurodegeneration in FA are the dentate nucleus of the cerebellum and the dorsal root ganglia of the spinal cord.[12] There is debate on the timing of damage to these structures relative to disease course, creating difficulty in determining the disease stage at which treatment of these structures would no longer rescue function.[13,14]

In addition to the challenges presented by treatment target, there are many other factors that may need consideration in gene therapy trial design for FA; particular factors include inclusion/exclusion criteria; length of trial; burden on patient/caregiver related to location of the trial and procedures; risks associated with dose, immunomodulation, and administration method; patient acceptance/aversion to these risks; patient perspective of disease burden; and availability of other trials. As a consequence, it is vital to consider the patient perspective in gene therapy clinical trial design.

Received 16 July 2023; accepted 13 December 2023;
https://doi.org/10.1016/j.omtm.2023.101179.

Correspondence: Manuela Corti, Department of Pediatrics, College of Medicine, University of Florida, Gainesville, FL 32611, USA.
E-mail: m.corti@peds.ufl.edu

© 2023 The Author(s).
This is an open access article under the CC BY license (http://creativecommons.org/licenses/by/4.0/).

Table 1. Demographics of the survey participants

Type of Respondent	N	%
Patient	88	64.23
Parent/caregiver	49	35.76

Fortunately, other disease communities undergoing gene therapy clinical trials, notably the Duchenne muscular dystrophy (DMD) community, have previously published research on understanding the patient perspective of gene therapy that provides an important foundation for our work. The first DMD study consisted of qualitative interviews that revealed how patients and caregivers felt about gene therapy, including risk acceptance and therapeutic expectations/priorities. The authors found through thematic analysis that these answers were influenced by the state of disease progression, with different therapeutic priorities and a higher level of risk acceptance associated with more severe disease.[15] The next DMD study was designed as a best–worst scaling questionnaire, asking participants to rank what they cared about most to least when choosing to participate in an early-phase gene therapy trial. The results indicated that participants prioritized potential benefits over potential harm and painful procedures.[16] Finally, Peay et al. designed a study in which gene therapy was described in a scenario as noncurative, with the effect of slowing progression for 10 years. Patients' and caregivers' maximum acceptable risk of mortality was then assessed for the initiation of gene therapy at different stages of disease progression. The authors discovered a relatively high tolerance for mortality risk overall that paralleled disease progression.[17]

Since FA has both shared and unique gene therapy trial design challenges, it is important to define the preferences specific to the FA community. This was designed as a descriptive pilot study to acquire results that could guide the design and development of future studies; however, we were also interested in testing the hypothesis that responses will differ between patients and parents/caregivers. This research is also crucial for the ongoing development of FA gene therapies. In a recent FDA Patient-Focused Drug Development listening meeting, the FDA expressed their desire for more formal research into patient perspectives on gene therapy and the incorporation of these data into trial design.[18]

RESULTS

Demographics

The summary statistics for the survey participants and the FA patients captured in the survey are outlined in Tables 1 and 2. For parent/caregiver responses, demographic information such as age and gender refers to the FA patient for whom the parent/caregiver is caring. Notably, the mean ages of onset and diagnosis do not accurately describe the sample due to a few higher-age outliers that create a skew in the distribution (Figure 1). The median ages of diagnosis (15 years) and onset (11 years) better describe our sample as representative of early-onset, or classical, FA.

Table 2. Demographics of the FA patients captured in the survey

	N	%	Mean	SD	Median
Male	62	45.26	–	–	–
Female	75	54.74	–	–	–
Current age, y	–	–	31.14	16.44	29
Age at diagnosis, y	–	–	18.92	11.81	15
Age at onset, y	–	–	15.01	11.62	11
Onset <18y	99	72.26	–	–	–
Onset ≥ 18	38	27.74	–	–	–

Symptoms

Participants were asked what their/the patient's first symptom was, which symptom most interferes with quality of life, and which symptom they would prefer to treat if only one could be treated. Patients with FA reported that their first symptom was balance/inability to walk (48.9%), impaired coordination (26.1%), or scoliosis (18.2%). Parents/caregivers of a patient with FA reported that their patient's first symptom was cardiomyopathy/heart problems (38.8%), balance/inability to walk (32.7%), or impaired coordination (28.6%). The distribution of responses for first symptom was significantly different between patients and parent/caregivers responding about their patient, $\chi^2(7) = 19.28$, $p < 0.01$. Quality of the patient's life was reported to be most affected by balance/inability to walk in 77.3% of self-reporting patients and 75.5% of parents/caregivers. Likewise, if only one symptom could be treated, then balance/inability to walk was chosen by 76.1% of patients and 71.4% of parents/caregivers. Although not statistically significant, it is notable that cardiomyopathy was chosen to be the treated symptom by 1.1% of patients and 10.2% of parents/caregivers (Figure 2).

Participant education

Before beginning the survey, participants were provided with a brief educational document that gave an overview of gene therapy and FA. A total of 71.6% of patients and 85.7% of caregivers agreed or strongly agreed that their knowledge of gene therapy improved after reviewing this educational introduction (Figure 3). The other participant education questions revealed that individuals with FA and caregivers feel knowledgeable about scientific advances in FA, different types of clinical trials, and gene therapy. Notably, there were significant differences in the level of agreement in responses from patients and parents/caregivers to the items "prior to the survey, I was knowledgeable about different types of clinical trials," $z = 2.35$, $p = 0.02$, and "I am aware of scientific advances in FA," $z = 3.17$, $p < 0.01$ (Figure 3).

The majority of participants reported that they learn about advances in FA research and upcoming clinical trials through patient advocacy organizations and the Internet. Less than half report learning this information from their healthcare providers (Figure 3).

Clinical trial perceptions

There were significant differences in the level of agreement in responses from patients and parents/caregivers to the items "I believe

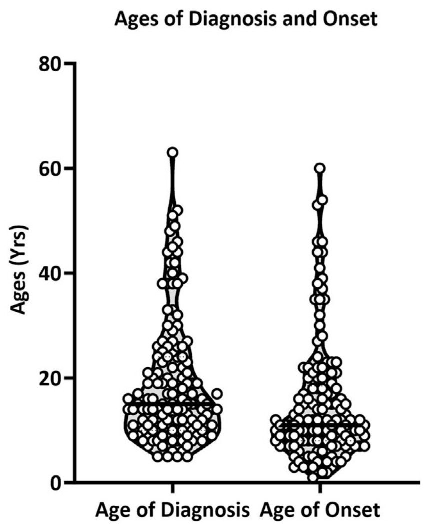

Figure 1. Ages of diagnosis and onset
This violin plot displays the age distribution of FA patients captured in the survey at disease onset and diagnosis.

clinical trials are important for scientific research," $z = 2.86$, $p < 0.01$, and "I believe clinical trials are necessary to study the treatment effects for FA," $z = 2.91$, $p < 0.01$ (Figure 4). There was also a significant difference in the level of agreement in responses from patients and parents/caregivers to the item "I believe that clinical trials for FA are focused on patient medical needs," $z = 2.27$, $p = 0.02$ (Figure 4).

A total of 49.4% of patients and 50.0% of parents/caregivers agree or strongly agree that patient preferences are incorporated in clinical trials for FA, whereas 76.7% of patients and 87.8% of parents/caregivers agree or strongly agree that they would be comfortable sharing these preferences about clinical trial study design (Figure 4). Lastly, 44.8% of patients and 52.1% of caregivers agree or strongly agree that they would be more likely to join a trial if researchers considered their preferences about the study design (Figure 4).

FA gene therapy perceptions

Respondents were first asked about their expectations of gene therapy for FA. For patients, 60.0% believe that gene therapy will reduce their symptoms, 59.5% anticipate that gene therapy will prevent their symptoms from worsening, and 25.0% believe that gene therapy is curative (Figure 5). For parents/caregivers, 63.3% believe that gene therapy will reduce symptoms, 67.4% anticipate that gene therapy will prevent symptoms from worsening, and 20.4% believe that gene therapy is curative (Figure 5).

There was a significant difference in the level of agreement in responses from patients and parents/caregivers to the item "I understand that gene therapy has risks," $z = 2.73$, $p \leq 0.01$ (Figure 5). Respondents were also asked whether they believe the most severe FA patients, mildly affected FA patients, or children should be treated first. There was a significant difference in the level of agreement in responses from patients and parents/caregivers to the item "I believe mildly affected patients with FA should be treated first," $z = 2.29$, $p = 0.02$, and no significant differences between respondents to the items "I believe the most severe patients with FA should be treated first" and "children with FA should be treated with gene therapy before adult patients" (Figure 5).

Factors influencing participation in gene therapy

Participants were asked a variety of questions about factors that may influence their participation in a gene therapy clinical trial. There were no significant differences in the level of agreement in responses from patients and parents/caregivers to these items. A total of 64.0% of patients and 59.0% of parents/caregivers neither agree nor disagree that they would prefer not to be treated with immunomodulation medicines in a gene therapy study (Figure 6). A total of 36.8% of patients and 40.8% of parents/caregivers agree or strongly agree that they would join a gene therapy study regardless of whether they were treated with immunomodulation medicines, and 49.4% of patients and 44.9% of parents/caregivers are ambivalent about this question (Figure 6). A total of 43.2% of patients and 49.0% of parents/caregivers neither agree nor disagree that the vector dose would not matter to them, whereas 35.2% of patients and 34.7% of parents/caregivers agree or strongly agree that the vector dose would not matter to them (Figure 6).

We then asked whether people would prefer to be treated immediately even if they would receive a lower dose, or if they preferred to be treated later if it meant they could receive a higher dose. A total of 46.6% of patients and 36.7% of parents/caregivers would prefer to be treated immediately even if it meant they would receive a lower dose, and 34.1% of patients and 51.0% of parents/caregivers were ambivalent (Figure 6). A total of 39.1% of patients and 30.6% of parents/caregivers would prefer to be treated later if they could receive a higher dose, and 43.7% of patients and 61.2% of parents/caregivers were ambivalent (Figure 6).

A total of 26.1% of patients and 12.2% of parents/caregivers would not join a gene therapy study if it meant they/their child could not receive gene therapy again in the future, whereas 44.3% of patients and 44.9% of parents/caregivers indicated that they would (Figure 6). Meanwhile, 45.5% of participants with FA and 39.6% of caregivers agreed or strongly agreed that they would join a gene therapy study regardless of the consequences and possible side effects (Figure 6).

Finally, we inquired about external factors that may influence the decision to participate; 37.5% of patients and 53.1% of parents/caregivers responded that the time commitment would not influence their decision to participate, whereas 36.8% of patients and 22.5% of parents/caregivers indicated that time commitment would be a factor. The availability of upcoming trials would be more influential as 60.2% of patients and 63.7% of parents/caregivers agreed or strongly agreed that would influence their decision to participate (Figure 6).

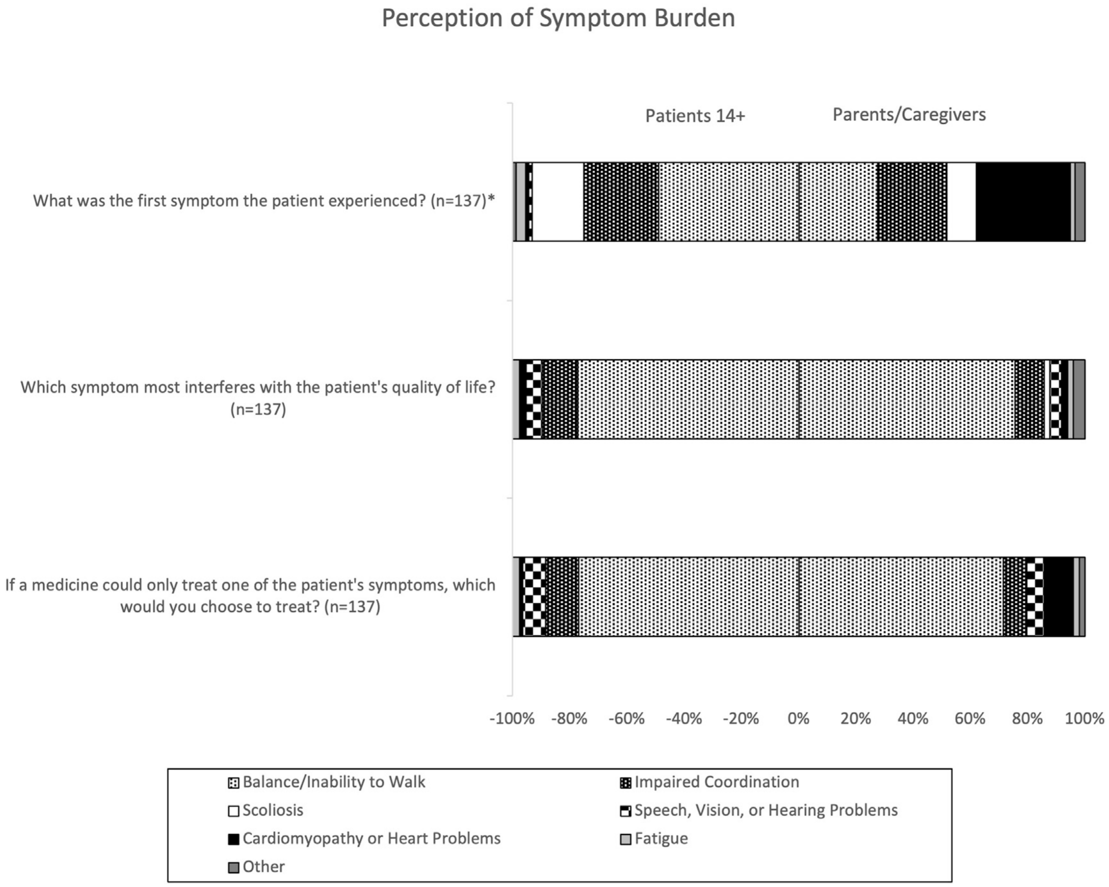

Figure 2. Perception of symptom burden

This 100% stacked bar chart represents participants' answers to 3 survey questions regarding symptom burden. The total number of respondents (n) is indicated next to each survey item. Items with a significantly different proportion of responses are indicated by an asterisk (*).

DISCUSSION

A strong majority of our sample reported feeling knowledgeable about gene therapy before the survey and having an increase in knowledge about gene therapy following reading the provided educational material in the survey introduction. Despite this perceived knowledge, the ambivalence about immunomodulation and the vector dose appears to indicate a lack of understanding about these topics. As we anticipated for this progressive disease with unmet medical need, there is a clear sense of urgency in obtaining gene therapy regardless of the consequences and possible side effects. We expected the proportion of respondents who would disregard risk to be higher among individuals with FA than caregivers of children with FA; however, there was no significant difference. Instead, we found a significant difference between patients and parents/caregivers in their understanding that gene therapy has risks. This raises the question of what risks patients and caregivers attribute to gene therapy.

The perceptions about FA gene therapy captured in our survey revealed high expectations for efficacy. Currently, there are no data in existence on the effect of gene therapy, for any tissue target, on human FA patients. Therefore, the spectrum of outcomes of gene therapy in this patient population are unknown. Our data suggest that people in the FA community are anticipating that gene therapy will have a substantial disease-modifying effect. This is an important finding for stakeholders who are creating patient education materials. It is also worth inquiring whether the high therapeutic expectations have contributed to the large proportion of respondents who would join a gene therapy study regardless of the consequences and possible side effects.

Furthermore, there is a considerable amount of ambivalence regarding whether the most severe FA patients, mildly affected FA patients, or children should be treated first. Respondents were generally in agreement that severe patients should be treated first. There was substantial uncertainty about whether children should be treated before adults. There was a significant difference in patient and parent/caregiver responses regarding whether mildly affected FA patients should be treated first. These responses may be due to the differences in the perceived risk of gene therapy and the high therapeutic expectations. The availability of the "neither agree nor disagree"

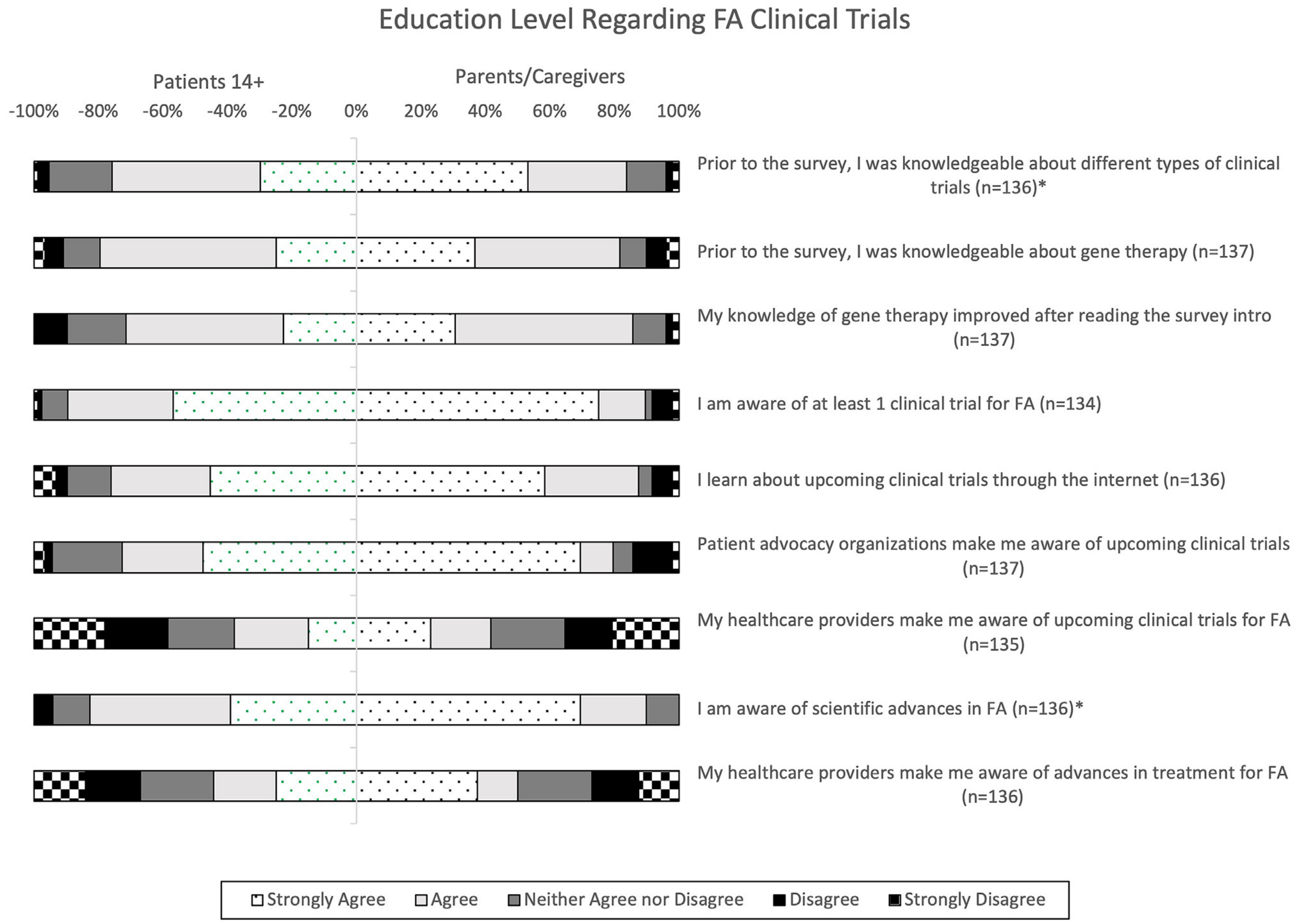

Figure 3. Participant education

This bidirectional 100% stacked bar chart represents patients' and caregivers' responses to survey questions regarding their education level on FA clinical trials. The proportions of patient responses are represented by the negative x axis and the proportions of caregiver responses are represented by the positive x axis. The total number of respondents (n) is indicated next to each survey item. Items with a significantly different proportion of responses are indicated by an asterisk (*).

option made it difficult to ascertain how participants really felt about who should be dosed first.

The patients' perceived symptom burden revealed another interesting finding. Cardiac complications present the most common cause of mortality in FA and are prevalent in childhood-onset classical FA, a population that is predominantly represented in this study. Despite this, respondents reported that their neurological symptoms most interfere with their quality of life and, overwhelmingly, that they would choose to treat these neurological symptoms over cardiac symptoms, if they could choose only one target symptom to treat. This is suggestive that individuals with FA prioritize an improved quality of life over an increased life expectancy. Although not significant, a higher proportion of caregivers than participants with FA would choose to treat cardiomyopathy, supporting the idea that patients may place higher value on quality of life over being alive. This conclusion is important for the prioritization of patient perspectives in research and drug development, especially because several FA gene therapies are in development with different treatment targets.[9] However, it is limited in that the survey did not include a question inquiring whether the patient has cardiac complications of FA. Although we can assume from the ages of onset and diagnosis that cardiac complications are present in the patients captured in the dataset, it could be possible that these patients do not experience cardiac complications. This could explain the decision not to treat it if they could only choose one symptom. Further research will be needed to explore this concept.

Participants reported that their decision to participate in a gene therapy clinical trial would be more strongly influenced by the options of other available and upcoming trials than by the time commitment required to participate in the trial. Many gene therapies or other treatments for FA are currently in development, which could increase the options of available and upcoming trials. Consequently, patients who meet the eligibility criteria for multiple trials will inevitably need to choose between participation in each of these trials. It is important to note that the time commitment will not be as heavy of an influence

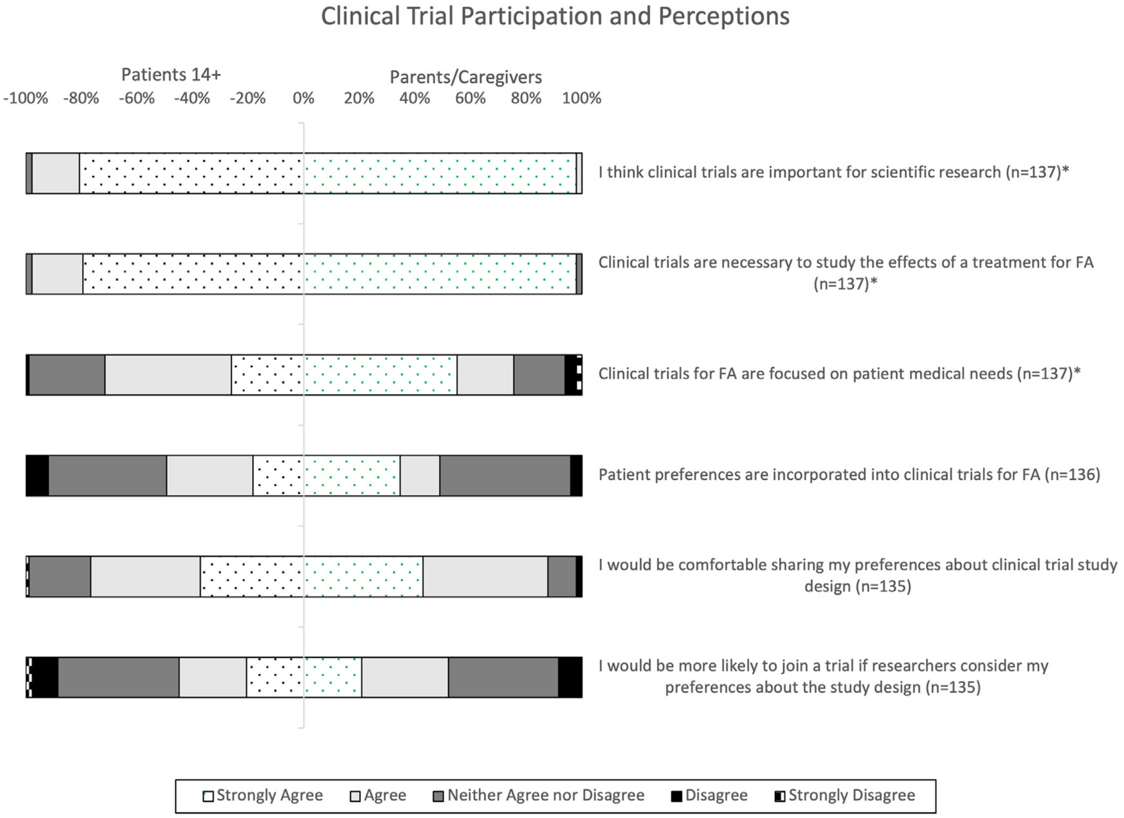

Figure 4. Clinical trial perceptions

This bidirectional 100% stacked bar chart represents patients' and caregivers' responses to survey questions regarding their clinical trial perceptions. The proportions of patient responses are represented by the negative x axis and the proportions of caregiver responses are represented by the positive x axis. The total number of respondents (n) is indicated next to each survey item. Items with a significantly different proportion of responses are indicated by an asterisk (*).

on the decision to participate since gene therapy trials will be longer than traditional clinical trials. This presents encouraging data that the increased length of gene therapy trials will not discourage participation.

It is interesting that although both patients and parents/caregivers strongly feel that "clinical trials are important and necessary" and "clinical trials for FA are focused on patient medical needs," parents/caregivers feel significantly more strongly about these statements than patients. Further investigation is required to explore these differences. The FA community is more ambivalent regarding whether patient preferences are incorporated into trials or whether this would affect their decision to join a trial; however, most participants would feel comfortable sharing their preferences, if asked.

Finally, our sample felt strongly that they are educated about scientific advances and upcoming clinical trials for FA. The Internet and patient advocacy organizations are more influential in providing this information to respondents than healthcare providers. This finding, however, is limited by the nature of recruitment (e-mail distribution via a patient organization's patient registry may bias the responses in favor of patient advocacy organizations). The nature of recruitment may also represent an overall limitation since it is unclear how representative the sample is of the overall population of the FA community in the United States. The mix of agreement and disagreement within both groups that healthcare providers provide information about upcoming clinical trials is interesting and may point to the fact that FA is a rare disease and, subsequently, there are a limited number of providers with clinical expertise in FA. These are relevant data for clinical trial recruitment.

In summary, the findings from this preliminary study of the FA community's perception of gene therapy trials offer insight into the education level of the community on gene therapy, their therapeutic expectations, and their perception of the disease burden. These data also highlight how the FA community obtains information about scientific advances in FA and upcoming clinical trials. Our study also indicates that the FA community would be comfortable sharing their preferences for clinical trial design. Lastly, this study identifies some differences in thought between patients and parents/caregivers. A potential limitation of our study is the inclusion of the educational document. It is unclear how any of the information in this document may have

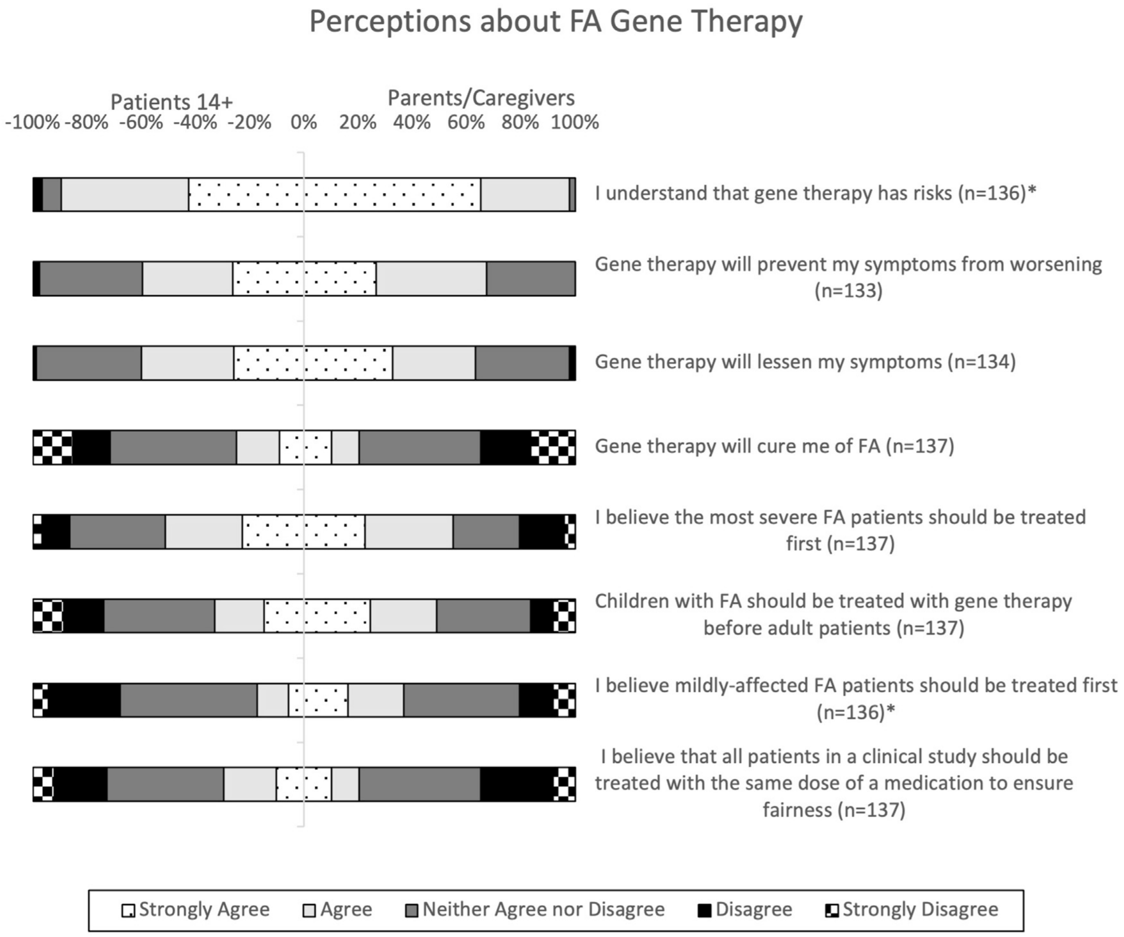

Figure 5. Gene therapy perceptions

This bidirectional 100% stacked bar chart represents patients' and caregivers' responses to survey questions regarding their FA gene therapy perceptions. The proportions of patient responses are represented by the negative x axis and the proportions of caregiver responses are represented by the positive x axis. The total number of respondents (n) is indicated next to each survey item. Items with a significantly different proportion of responses are indicated by an asterisk (*).

influenced survey responses, if at all. Our findings represent important information for stakeholders regarding clinical trial design and community education, which is crucial for patient-focused drug development.

MATERIALS AND METHODS

This cross-sectional study was designed at the University of Florida and approved by the institutional review board (IRB). Individuals were recruited for this study by 2 e-mails sent on March 8, 2022 and March 22, 2022 to 470 individuals in the United States through the Friedreich's Ataxia Global Patient Registry (FAGPR). The FAGPR is a database that collects information on FA patients and distributes recruitment notices for clinical trials and studies to eligible individuals. Involvement in the FAGPR is entirely voluntary, and receipt of a recruitment notice does not mandate participation. The educational document was attached to the recruitment e-mail. This document gave an overview of FA, gene therapy, immunomodulation, and the differences between nongene therapy and gene therapy clinical trials. Copies of the IRB approval letter, recruitment e-mail, and educational document are available in the supplemental information.

Individuals interested in voluntary participation were directed to the IRB-approved informed consent. Participants were instructed to read the consent in its entirety. At the bottom of the page, the participants were instructed to electronically consent to participate via "Yes" or "No" selections. Due to the required anonymity of the study, participants did not indicate their name nor their location on the consent, meaning there were no screening mechanisms. Following agreement, the participant was instructed to move to the next page to begin the questionnaire. Only once consent/assent was obtained could the participant continue to the questionnaire. Participants were electronically provided with a copy of the consent document for their personal records.

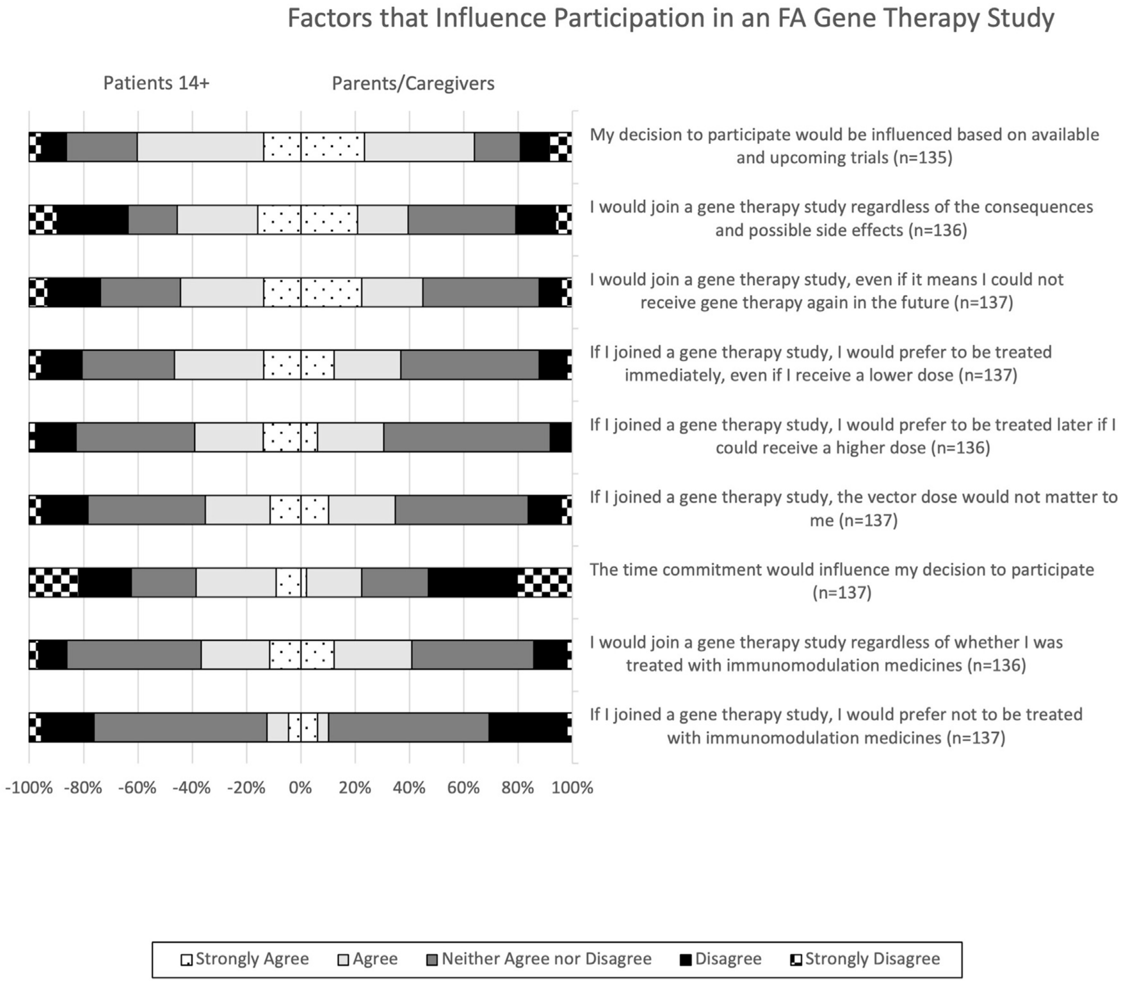

Figure 6. Factors influencing participation in gene therapy

This bidirectional 100% stacked bar chart represents patients' and caregivers' responses to survey questions regarding the factors that would influence their participation in a gene therapy clinical trial. The proportions of patient responses are represented by the negative x axis and the proportions of caregiver responses are represented by the positive x axis. The total number of respondents (n) is indicated next to each survey item. There are no items with significantly different proportions of responses.

The survey was conducted through the Internet using the browser-based, electronic data collection service REDcap between March 8, 2022 and April 18, 2022. The survey included a few questions about age, diagnosis, onset, and symptoms. Participants then answered Likert-style questions ranging from "strongly agree" to "strongly disagree" regarding a variety of topics related to gene therapy and clinical trials. A full copy of the survey is available in the supplemental information.

The directions stated that the following individuals could participate in the survey: parents/caregivers of children with FA younger than 14 years, patients 14 years and older, and parents/caregivers of children with FA aged 14–17 years collaboratively with their children. A total of 156 records were saved; however, 15 of these were incomplete. Thus, 141 complete records were included for preprocessing. This is a response rate of 30.0%. All of the patient records came from patients with a current age of 14 years or older, as intended by the directions. A total of 35% (18/52) of the parent/caregiver records came from parents/caregivers of a patient currently 18 years or older, which was not indicated by the directions. Rather than excluding this large proportion of data, the category for analysis was adjusted to include parent/caregivers of patients who were diagnosed with FA as a child (regardless of their current age). Thus, we only excluded 4 complete records, which were from parent/caregivers of patients who were diagnosed with FA as adults. The final number of records for analysis was 137. Data quality was reviewed for validity by 3 authors and an external contributor.

Data analysis

Summary statistics were generated using Microsoft Excel, and figures were generated using Microsoft Excel and GraphPad Prism. For statistical analysis, participants were divided into two comparison

groups in terms of the question "who is completing this survey?": patients with FA and parents/caregivers of an individual with FA who was diagnosed as a child. For the questions with nominal categorical responses, χ^2 exact tests were performed to detect the differences between the two groups. For the questions with Likert scale responses, items were first converted into an ordinal scale and Mann-Whitney tests were then performed. Items with a $p < 0.05$ were considered significant. Statistical analysis was implemented with SAS (version 9.4, SAS Institute, Cary, NC).

DATA AND CODE AVAILABILITY

All of the data generated or analyzed during this study are included in the published article.

SUPPLEMENTAL INFORMATION

Supplemental information can be found online at https://doi.org/10.1016/j.omtm.2023.101179.

ACKNOWLEDGMENTS

We thank the FA community for participating in this study, as well as FARA for reviewing and providing feedback on the educational material and survey, and for distributing the survey through the patient registry. We also thank M. Donepudi, L. Wilkinson, and C. Wright for their recommendations regarding the initial survey design. Alexander Simon (ClinicalMind, New York, NY) provided graphical support for the educational material under the direction of M.C. Megan P. Wolf provided significant support for the creation of the graphical abstract and reviewed the data for validity. This study was funded by departmental funding at the University of Florida (to M.C.). AavantiBio supported the work at ClinicalMind.

AUTHOR CONTRIBUTIONS

M.C. and E.C. designed the survey and educational material. M.A.C., S.J.T., S.N., J.B., S.S., and B.J.B. reviewed and provided edits on the survey questions. M.C., S.J.T., and M.A.C. reviewed and analyzed the data, and X.L. performed statistical analysis. S.J.T. wrote the first draft of the manuscript. All of the authors agreed on the content of the manuscript, reviewed the draft, and approved the final version.

DECLARATION OF INTERESTS

M.C. and B.J.B. are co-founders of AavantiBio, which supported the employee at ClinicalMind who provided graphical support for the educational material written by the study team. The survey was completed before the acquisition of AavantiBio by Solid Biosciences. None of the authors have any financial, commercial, legal, or professional relationship with Solid Biosciences. There was no commercial support for this study. The study was developed and conducted by the academic team at the University of Florida. Thus, the authors declare that they have no competing interests.

REFERENCES

1. Pandolfo, M. (2009). Friedreich ataxia: the clinical picture. J. Neurol. *256* (*Suppl 1*), 3–8.
2. Jiralerspong, S., Liu, Y., Montermini, L., Stifani, S., and Pandolfo, M. (1997). Frataxin Shows Developmentally Regulated Tissue-Specific Expression in the Mouse Embryo. Neurobiol. Dis. *4*, 103–113.
3. Ohshima, K., Montermini, L., Wells, R.D., and Pandolfo, M. (1998). Inhibitory effects of expanded GAA·TTC triplet repeats from intron I of the Friedreich ataxia gene on transcription and replication in vivo. J. Biol. Chem. *273*, 14588–14595.
4. Labuda, M., Labuda, D., Miranda, C., Poirier, J., Soong, B.W., Barucha, N.E., and Pandolfo, M. (2000). Unique origin and specific ethnic distribution of the Friedreich ataxia GAA expansion. Neurology *54*, 2322–2324.
5. Geoffroy, G., Barbeau, A., Breton, G., Lemieux, B., Aube, M., Leger, C., and Bouchard, J.P. (1976). Clinical Description and Roentgenologic Evaluation of Patients with Friedreich's Ataxia. Can. J. Neurol. Sci. *3*, 279–286.
6. Friedman, L.S., Farmer, J.M., Perlman, S., Wilmot, G., Gomez, C.M., Bushara, K.O., Mathews, K.D., Subramony, S.H., Ashizawa, T., Balcer, L.J., et al. (2010). Measuring the rate of progression in Friedreich ataxia: Implications for clinical trial design. Mov. Disord. *25*, 426–432.
7. Payne, R.M. (2022). Cardiovascular Research in Friedreich Ataxia: Unmet Needs and Opportunities. JACC Basic Transl. Sci. *7*, 1267–1283.
8. Tsou, A.Y., Paulsen, E.K., Lagedrost, S.J., Perlman, S.L., Mathews, K.D., Wilmot, G.R., Ravina, B., Koeppen, A.H., and Lynch, D.R. (2011). Mortality in Friedreich Ataxia. J. Neurol. Sci. *307*, 46–49.
9. (2023). The Food & Drug Administration. https://www.fda.gov/drugs/news-events-human-drugs/fda-approves-first-treatment-friedreichs-ataxia.
10. Friedreich's Ataxia Research Alliance. https://www.curefa.org/research/research-pipeline.
11. Zhou, K., Han, J., Wang, Y., Zhang, Y., and Zhu, C. (2022). Routes of administration for adeno-associated viruses carrying gene therapies for brain diseases. Front. Mol. Neurosci. *15*, 988914.
12. Koeppen, A.H., and Mazurkiewicz, J.E. (2013). Friedreich ataxia: Neuropathology revised. J. Neuropathol. Exp. Neurol. *72*, 78–90.
13. Koeppen, A.H., Becker, A.B., Qian, J., Gelman, B.B., and Mazurkiewicz, J.E. (2017). Friedreich Ataxia: Developmental Failure of the Dorsal Root Entry Zone. J. Neuropathol. Exp. Neurol. *76*, 969–977.
14. Rezende, T.J.R., Martinez, A.R.M., Faber, I., Girotto Takazaki, K.A., Martins, M.P., de Lima, F.D., Lopes-Cendes, I., Cendes, F., and França, M.C., Jr. (2019). Developmental and neurodegenerative damage in Friedreich's ataxia. Eur. J. Neurol. *26*, 483–489.
15. Landrum Peay, H., Fischer, R., Tzeng, J.P., Hesterlee, S.E., Morris, C., Strong Martin, A., Rensch, C., Smith, E., Ricotti, V., Beaverson, K., et al. (2019). Gene therapy as a potential therapeutic option for Duchenne muscular dystrophy: A qualitative preference study of patients and parents. PLoS One *14*, e0213649.
16. Paquin, R.S., Fischer, R., Mansfield, C., Mange, B., Beaverson, K., Ganot, A., Martin, A.S., Morris, C., Rensch, C., Ricotti, V., et al. (2019). Priorities when deciding on participation in early-phase gene therapy trials for Duchenne muscular dystrophy: a best-worst scaling experiment in caregivers and adult patients. Orphanet J. Rare Dis. *14*, 102.
17. Peay, H.L., Fischer, R., Mange, B., Paquin, R.S., Smith, E.C., Sadosky, A., Russo, L., Ricotti, V., Rensch, C., Morris, C., et al. (2021). Patients' and caregivers' maximum acceptable risk of death for non-curative gene therapy to treat Duchenne muscular dystrophy. Mol. Genet Genomic Med. *9*, e1664.
18. (2023). FDA CBER OTAT Patient Focused Drug Development Listening Meeting Summary Report. https://www.fda.gov/media/168183/download.

OMTM, Volume *32*

Supplemental information

Perspectives of the Friedreich ataxia community on gene therapy clinical trials

Shandra J. Trantham, Mackenzi A. Coker, Samantha Norman, Emma Crowley, Julie Berthy, Barry J. Byrne, Sub Subramony, XiangYang Lou, and Manuela Corti

Email to Potential Participants:

Subject: Invitation to participate in FA Gene Therapy Survey

FARA is sharing information about a survey that is evaluating opinions about gene therapy. The results of this survey will help inform researchers, physicians, and biopharmaceutical companies about the FA community's knowledge and opinions on gene therapy. This information may also be utilized to guide future research and education for the FA community.

WHO CAN PARTICIPATE?

An individual living with FA or as a parent/guardian of a child living with FA and are a US resident.

- Individuals who complete the survey must be 14 years of age or older.
  - *Parents of children ages 14 to 17 years old, may elect to complete the survey together.*
- If the child is less than 14 years old, we ask that a parent complete the survey for them.

WHAT'S INVOLVED?

We ask you to read information about the survey, provide online consent, view a PowerPoint that has introductory information and then complete of an online survey. The online survey includes questions in regards to demographic and FA symptom and asks about your preferences and opinions related to gene therapy. The survey will take approximately 20 minutes to complete and all responses will be strictly confidential. The survey answers will be completely anonymous. Participants can skip any questions they do not wish to answer.

If you wish to participate in this survey, please click this link to participate:
https://redcap.ctsi.ufl.edu/redcap/surveys/?s=8L8XLYDXNJ

MORE INFORMATION:

If you have any questions or comments about this survey, please contact Mackenzi Coker. You can reach her at [REDACTED] or [REDACTED].

Thank you for considering participating in this important study.

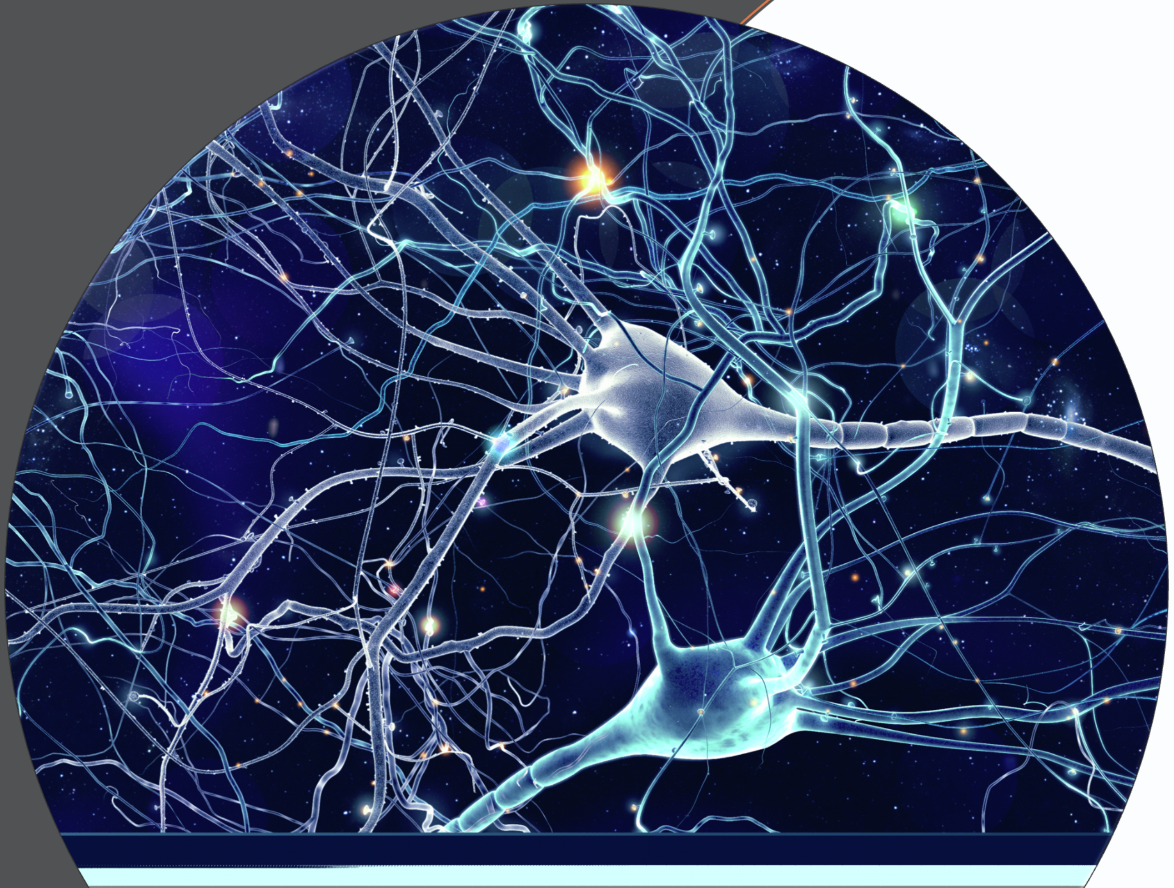

This study is a collaboration between UF and AavantiBio

Friedreich's Ataxia Gene Therapy Questionnaire

Introduction

UF UNIVERSITY of FLORIDA

AAVANTIBIO

Introduction

Thank you for choosing to participate in this survey. This survey is being distributed through patient advocacy groups for Friedreich's ataxia (FA). The purpose is to collect opinions about gene therapy research for FA. Patients with FA or their family members may take the survey. The results will be used to help develop future clinical studies for FA.

If you are 14 years old or younger, please ask a parent or caregiver to complete the survey for you. Before answering the questions, please review this PowerPoint and read the additional information from the link.

FA and Gene Therapy

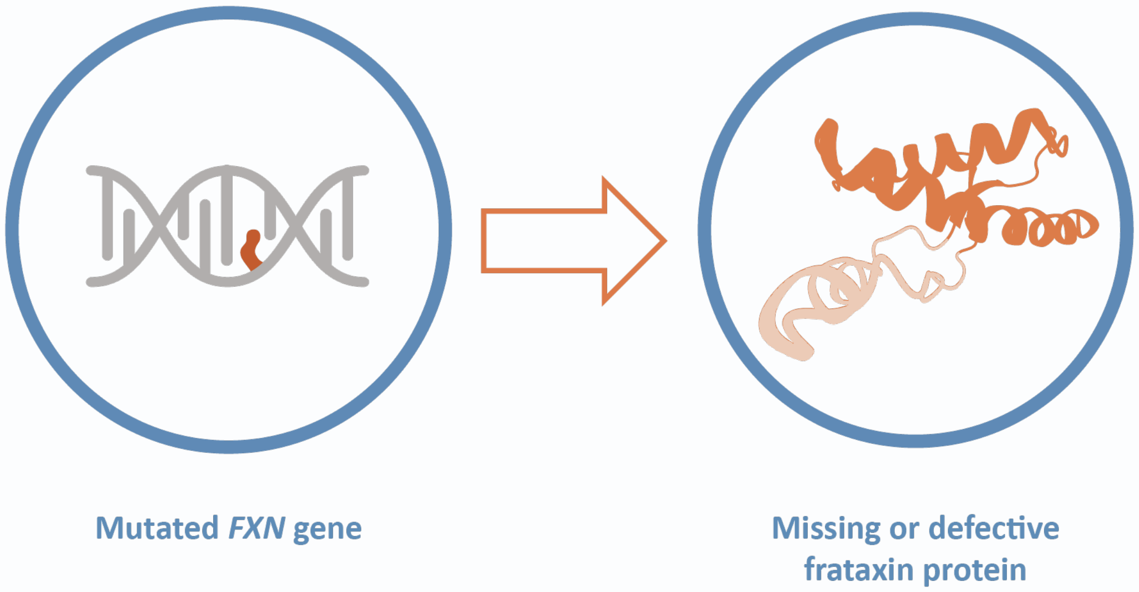

FA is a rare disease that is caused by defects (mutations) in the gene for a protein called frataxin. These mutations interfere with the production or function of frataxin. Lack of frataxin leads to the symptoms of FA.

UF UNIVERSITY of FLORIDA AAVANTIBIO

FA and Gene Therapy

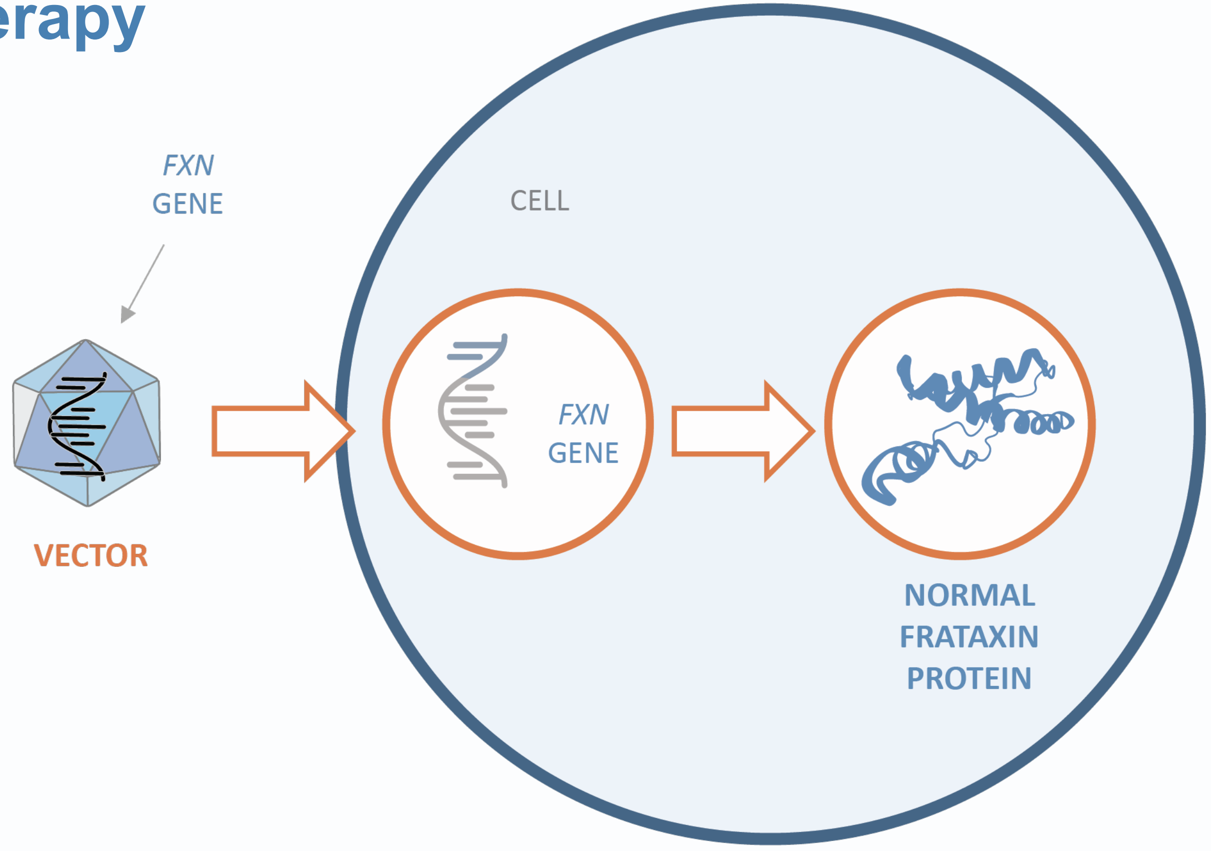

Gene therapy is a potential treatment for FA. Gene therapy works by putting new genes for frataxin into cells to restore function. The genes provide instructions for making frataxin protein. Increasing the amount of frataxin in cells may help reduce the severity of FA. These genes are delivered by a vehicle called a vector. The vector is a modified virus that does not cause any disease.

UF UNIVERSITY of FLORIDA AAVANTIBIO

FA and Gene Therapy

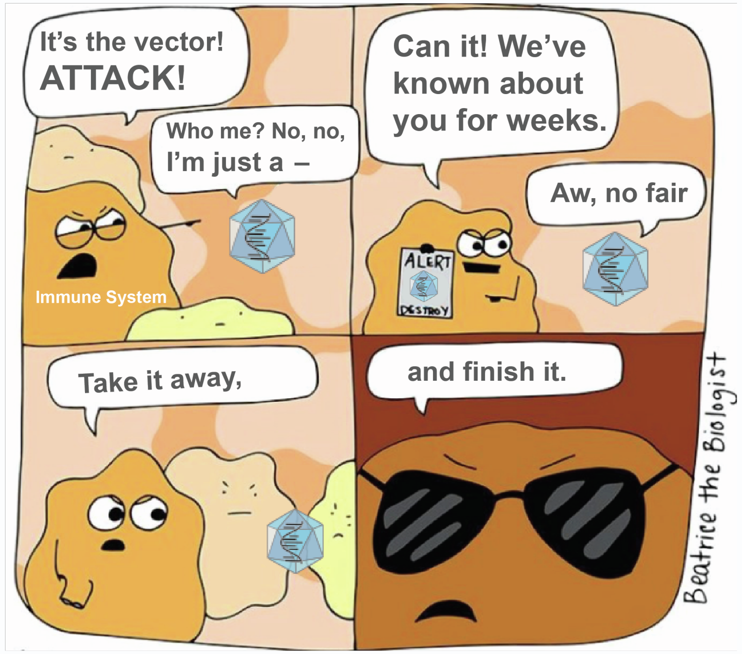

Currently, patients who receive gene therapy can only be treated once. This is because the body's defense system (immune system) remembers the vector. If it sees the vector again, it will attack the vector and prevent it from working.

Researchers are trying to find ways to prevent this from happening. If they are successful, patients may be able to receive gene therapy more than once. This may help gene therapy last longer. It could also allow patients to participate in future studies (clinical trials) on gene therapy.

Figure adapted from McKissick K. Get Your Flu Shot. Beatrice the Biologist. November 7, 2014. Accessed November 9, 2021. www.beatricebiologist.com/2014/

UF UNIVERSITY of FLORIDA AAVANTIBIO

FA and Gene Therapy

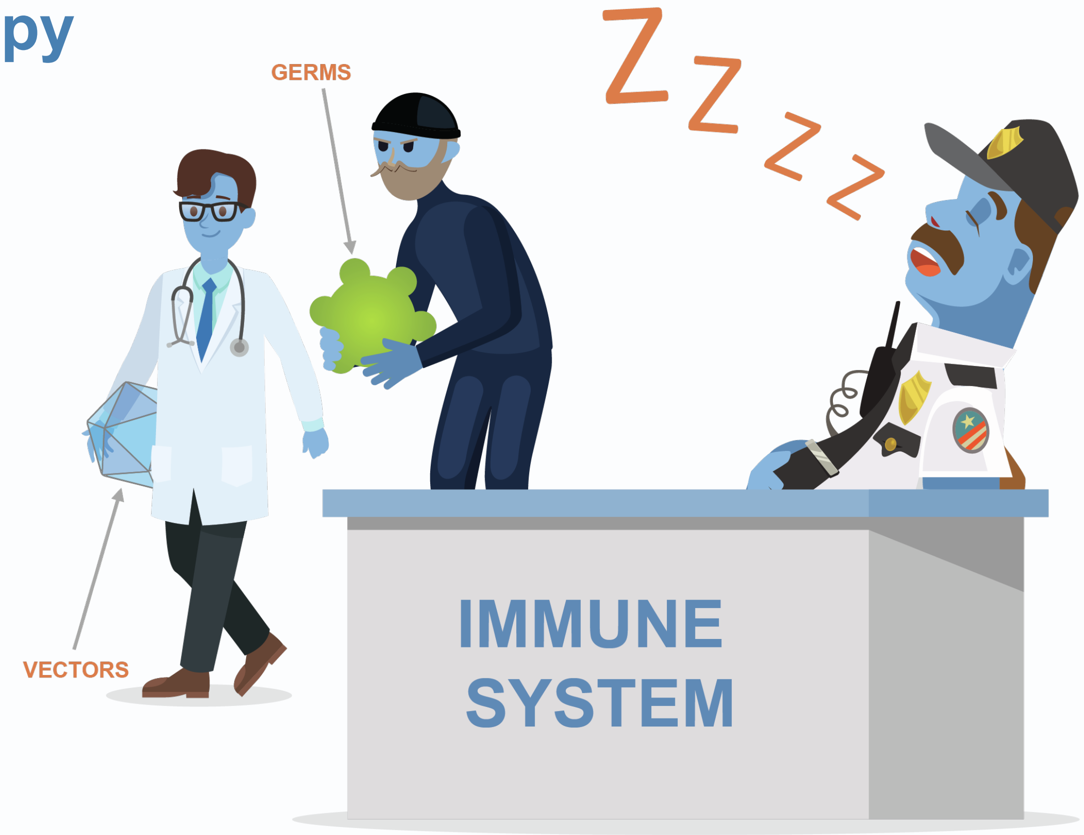

Researchers are testing medicines that change how the immune system works. This is called immunomodulation. Immunomodulation medicines could be used with gene therapy. Then the immune system would not attack the vector. However, immunomodulation medicines may make common illnesses, such as a cold or flu, more severe than usual. This is why doctors and nurses closely monitor patients who are treated with these medicines.

Figure adapted from media.istockphoto.com/vectors/guardpoliceman-napping-vector-id120881647

UF | UNIVERSITY of FLORIDA   AAVANTIBIO

Gene Therapy and Clinical Trials

Placebo-Controlled Clinical Trial

Researchers perform clinical trials to test whether medicines can improve people’s health.

There are different kinds of clinical trials. One is called a placebo-controlled study. Participants are divided into 2 groups. One group is given an inactive, harmless treatment (placebo). The other group is treated with the new medicine.

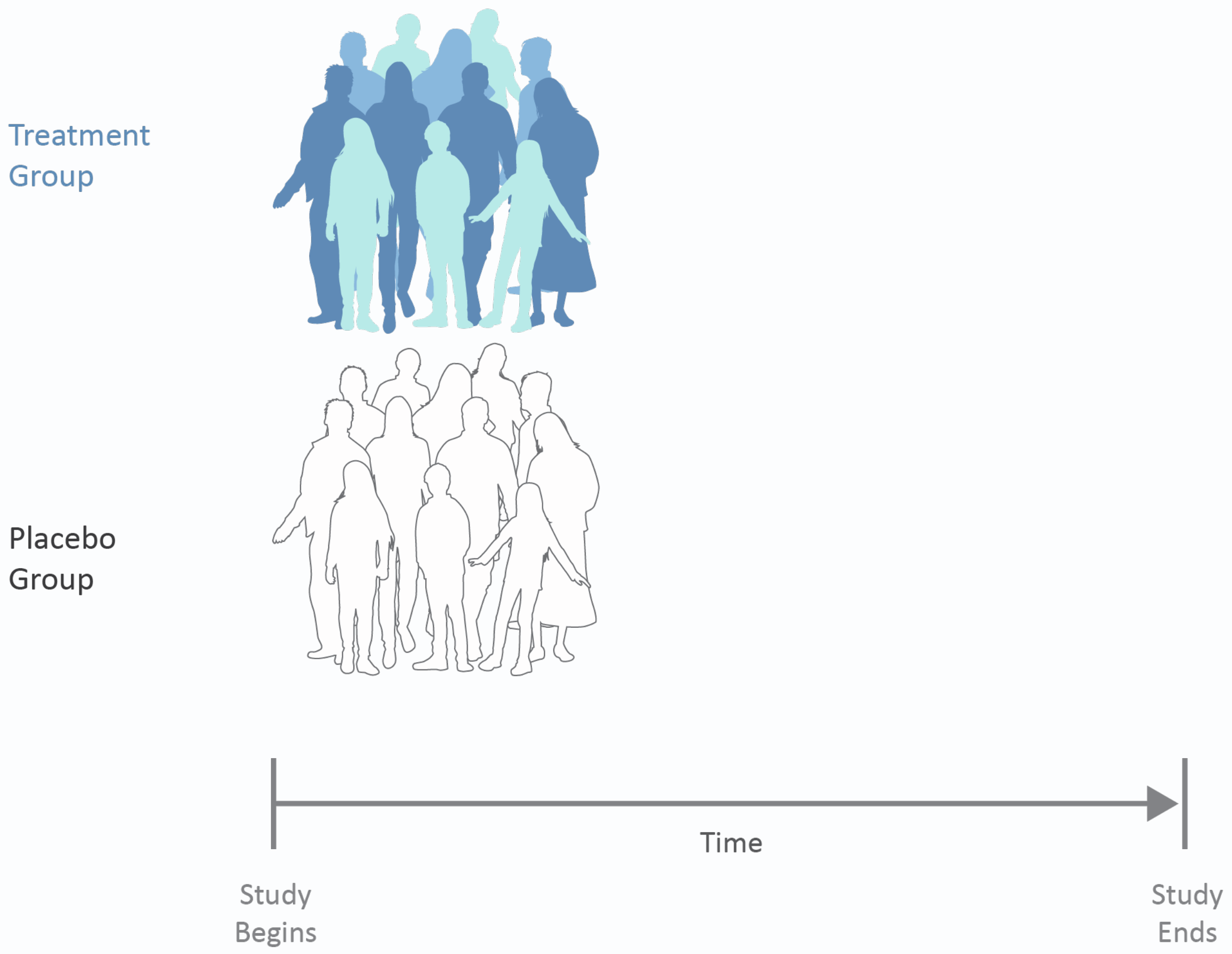

UF UNIVERSITY of FLORIDA

AAVANTIBIO

Gene Therapy and Clinical Trials

Delayed-Treatment Clinical Trial

Because of the potential benefit, trial designs try to minimize size and length of placebo. In some cases , researchers can perform a delayed-treatment clinical trial. Participants are divided into 2 groups. One group receives the potential new medicine immediately. The placebo group receives the same active treatment after a period of time.

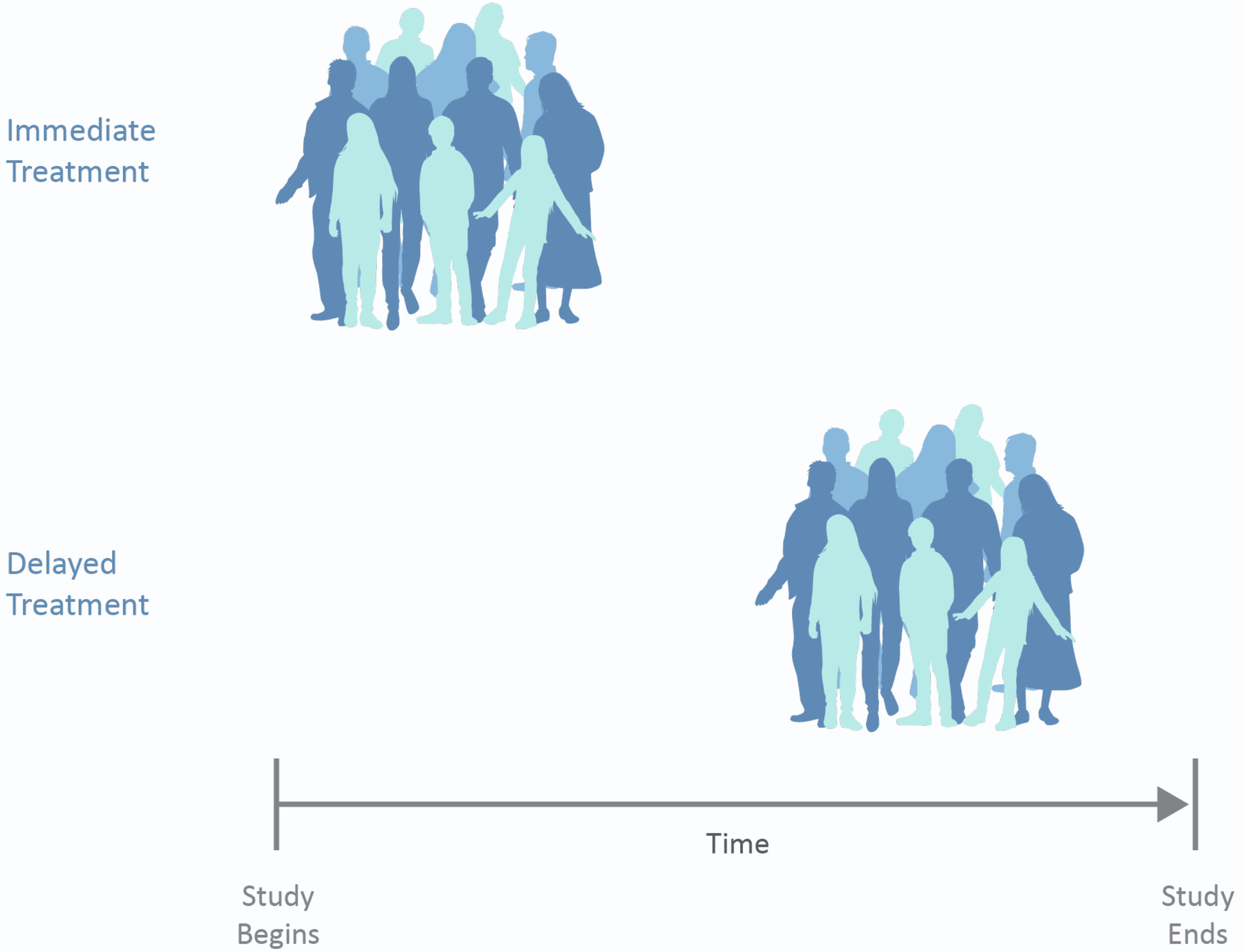

UF | UNIVERSITY of FLORIDA

AAVANTIBIO

Back to RedCap

Now that you have reviewed the FA Questionnaire Introduction, please head back to the RedCap link to read the additional information and complete the survey, if you agree to participate.

UF | UNIVERSITY of FLORIDA   AAVANTIBIO

Title of Project: Friedreich's Ataxia Gene Therapy Questionnaire

Principal Investigator: Manuela Corti, Assistant Professor
Powell Center for Rare Disease Research and Therapy, University of Florida

1. **Purpose of the Study:** This survey collects opinions about gene therapy research for Friedreich's ataxia (FA). Patients with FA or their family members may take the survey.
2. **Instructions to be Followed:** You will be asked to read a 1-page introduction and answer 42 questions.
3. **Duration:** The survey will take about 15 minutes.
4. **Benefits of Participating:** There is no direct benefit to you. The survey results may benefit others.
5. **Risks of Participating:** There is minimal risk.
6. **Confidentiality:** Your participation in this survey is confidential. Your responses are not linked to your name. If the survey results are published, researchers will not identify you.
7. **Right to Ask Questions:** Please call Mackenzi Coker at 352-294-8754 if you have any questions or concerns about this study.
8. **Privacy Authorization**: University of Florida and Shands Hospital can only share your medical information with the following people:
    a. Other researchers conducting approved research
    b. The sponsor of a research study
    c. United States government agencies
9. **Voluntary Participation:** Your decision to participate in this survey is voluntary. You can stop at any time. You may skip any question that you do not want to answer. If you choose not to participate, this will not affect your current or future healthcare at Shands Hospital or University of Florida. If you have any questions about your rights as a participant, please call the Institutional Review Board at 352-273-9600.

Completion of the survey means that you have read the information above. It also means that you agree to take part in the study. Please keep this information for your records.

CONFIDENTIAL

Thank you for choosing to participate in this survey. This survey is being distributed through patient advocacy groups for Friedreich's ataxia (FA). The purpose is to collect opinions about gene therapy research for FA. Patients with FA or their family members may take the survey. The results will be used to help develop future clinical studies for FA.

If you are 14 years old or younger, please have a parent or caregiver complete the survey for you. Before answering the questions, please download the Introductory PowerPoint and then read the information below.

Please ensure you have reviewed the Introductory PowerPoint, prior to reading the information below.

FA and Gene Therapy

FA is a rare disease that is caused by defects (mutations) in the gene for a protein called frataxin. These mutations interfere with the production or function of frataxin. Lack of frataxin leads to the symptoms of FA.

Gene therapy is a potential treatment for FA. Gene therapy works by inserting new genes for frataxin into cells to restore function. The genes provide instructions for making frataxin protein. Increasing the amount of frataxin in cells may help reduce the severity of FA. These genes are delivered by a vehicle called a vector. The vector is a modified virus that does not cause any disease.

Currently, patients who receive gene therapy can only be treated once. This is because the body's defense system (immune system) remembers the vector. If it sees the vector again, it will attack the vector and prevent it from working. Researchers are trying to find ways to prevent this from happening. If they are successful, patients may be able to receive gene therapy more than once. This may help gene therapy last longer. It could also allow patients to participate in future studies (clinical trials) on gene therapy.

In addition to the body potentially attacking the vector, there are other potential side effects / risks that have been observed in human gene therapy trials. Some of those side effects include, but are not limited to: Nausea and vomiting, low platelet counts, fever, allergic reactions, liver inflammation, tissue injury, headache, generalized inflammation, cardiac problems, and abnormal blood tests. Please keep in mind that gene therapy research remains relatively new, therefore, all side effects / risks are not known. In (very) rare cases side effects can be severe and lead to death. Researchers are testing medicines that change how the immune system works. This is called immunomodulation. Immunomodulation medicines could be used with gene therapy. Then the immune system would not attack the vector, and could help reduce the risk / alleviate some of the known side effects. However, immunomodulation medicines may make common illnesses, such as a cold or flu, more severe than usual. This is why doctors and nurses closely monitor patients who are treated with these medicines.

Gene Therapy and Clinical Trials

It is anticipated that gene therapy will improve a patient's condition. When tested in other conditions the patient's condition has greatly improved.

Researchers perform clinical trials to test whether medicines are safe and can improve people's health. There are different kinds of clinical trials. One is called a placebo-controlled study. Participants are divided into 2 groups. One group is given an inactive, harmless treatment (placebo). The other group is treated with the potential new medicine.

Because of the potential benefit, trial designs try to minimize size and length of placebo. In some cases, researchers can perform a delayed-treatment clinical trial. Participants are divided into 2 groups. One group receives the potential new medicine immediately. The placebo group receives the same active treatment after a period of time. However some participants in the placebo group may become ineligible by the time they are able to receive the medicine.

Some clinical trials have more than 1 treatment group. Researchers may compare different amounts (doses) of the potential new medicine. This is called a dose-escalation study. In a dose-escalation study, a lower dose is studied

CONFIDENTIAL

first. If the lower dose is safe, a higher dose may be tested next. This type of clinical trial helps researchers determine which dose works best and is safest. A higher dose may be more effective but may have more side effects.

Consent to Participate in This Study

Thank you for choosing to participate in this survey. If you are 14 years old or younger, please ask a parent or caregiver to complete the survey for you. Your participation is voluntary. You do not have to answer any questions that you do not want to. You may stop the survey at any time. All answers will be kept anonymous. There is minimal risk with your participation. Please indicate below if you are willing to participate.

☐ I have read and understand the information above to the best of my ability. I am aware that my participation is voluntary. I understand that my answers are not linked to my name.

If you have any questions or comments about this survey, please contact Mackenzi Coker. You can reach her at mcoker@peds.ufl.edu or 352-294-8754.

Please continue to the next page to begin the survey.

Please answer the questions below as accurately as possible.

1) Who is completing this survey? (Choose one)

Parent/Caregiver of patient with Friedreich's ataxia (FA) ☐ Patient with FA ☐

2) What is your/the patient's current age? ___________

3) What is your/the patient's gender?

Male ☐ Female ☐ Non-Binary ☐ Prefer not to answer ☐

4) How old were you/was the patient at the time of diagnosis? __________

5) How old were you/was the patient when symptoms were first noticed? __________

6) What was the first symptom you/the patient experienced? This includes symptoms experienced prior to diagnosis. (Choose one)

Balance/Inability to Walk	☐
Impaired Coordination	☐
Fidgetiness	☐
Fatigue	☐
Scoliosis (abnormal curvature of the spine)	☐
Cardiomyopathy or Heart Problems	☐
Speech, Vision, or Hearing Problems	☐
Other (Please specify:)	☐

7) Which of the following symptoms most interferes with your/the patient's quality of life today? (Choose one) (Choose one)

Balance/Inability to Walk	☐
Impaired Coordination	☐

CONFIDENTIAL

Fidgetiness ☐
Fatigue ☐
Scoliosis (abnormal curvature of the spine) ☐
Cardiomyopathy or Heart Problems ☐
Speech, Vision, or Hearing Problems ☐
Other (Please specify:) ☐

8) If a medicine could treat only one of your/the patient's symptoms, which would you/the patient prefer? (Choose one)

Balance/Inability to Walk ☐
Impaired Coordination ☐
Fidgetiness ☐
Fatigue ☐
Scoliosis (abnormal curvature of the spine) ☐
Cardiomyopathy or Heart Problems ☐
Speech, Vision, or Hearing Problems ☐
Other (Please specify:) ☐

9) Have you/Has the patient previously participated in any clinical trial?

Yes ☐ No ☐

10) Please share your/the patient's opinions about the following statements. (Choose one option in each row.)

	Strongly Disagree	Disagree	Neither Agree nor Disagree	Agree	Strongly Agree
10.1. I think clinical trials are important for scientific research.					
10.2. Clinical trials are necessary to study the effects of treatments for FA.					
10.3. I am aware of scientific advances in FA.					
10.4. My healthcare providers make me aware of advances in treatment for FA.					
10.5. My healthcare providers make me aware of upcoming clinical trials for FA.					
10.6. Patient advocacy organizations for FA make me aware of upcoming clinical trials.					
10.7. I learn about upcoming clinical trials through the internet.					
10.8. I am aware of at least 1 clinical trial for FA.					
10.9. Clinical trials for FA are focused on patient medical needs ~~needs~~.					

CONFIDENTIAL

10.10. Patient preferences ~~opinions~~ are incorporated into clinical trials for FA.					

11) Prior to this survey, had you/the patient heard about gene therapy?

Yes ☐ No ☐ I don’t know ☐

12) Please share your/the patient’s opinions about the following statements. (Choose one option in each row.)

	Strongly Disagree	Disagree	Neither Agree nor Disagree	Agree	Strongly Agree
12.1. Prior to this survey, I was knowledgeable about gene therapy.					
12.2. Prior to this survey, I was knowledgeable about different types of clinical trials.					
12.3. My knowledge of gene therapy improved after reading the survey introduction.					
12.4. Gene therapy will lessen my symptoms.					
12.5. Gene therapy will prevent my symptoms from worsening.					
12.6. Gene therapy will cure me of FA.					
12.7. I would be more likely to join a clinical trial if researchers consider my preferences about the study design.					
12.8. I would be comfortable sharing my preferences about clinical trial study design.					
12.9. I understand that gene therapy has risks.					

CONFIDENTIAL

13) Please share your/the patient's opinions about joining a gene therapy clinical trial. (Choose one option in each row.)

	Strongly Disagree	Disagree	Neither Agree nor Disagree	Agree	Strongly Agree
13.1. I would join a gene therapy study, even if it means I could not receive gene therapy again in the future.					
13.2. I would join a gene therapy study regardless of the consequences and possible side effects.					
13.3. I believe the most severe patients with FA should be treated first.					
13.4. I believe mildly affected patients with FA should be treated first.					
13.5. Children with FA should be treated with gene therapy before adult patients.					
13.6. If I joined a gene therapy study, I would prefer **not** to be treated with immunomodulation medicines.					
13.7. I would join a gene therapy study regardless of whether I was treated with immunomodulation medicines.					
13.8. If I joined a gene therapy study, the vector dose would not matter to me.					
13.9. If I joined a gene therapy study, I would prefer to be treated immediately, even if I receive a lower dose.					
13.10. If I joined a gene therapy study, I would prefer to be treated later if I could receive a higher dose.					
13.11. I believe that all patients in a clinical study should be treated with the same dose of a medication to ensure fairness.					
13.12. The time commitment would influence my decision to participate.					
13.13. My decision to participate would be influenced based on available and upcoming trials.					

CONFIDENTIAL

Health Center Institutional Review Board
FWA00005790

PO Box 100173
Gainesville FL 32610-0173
Telephone: (352) 273-9600
Facsimile: (352) 273-9614
Email: irb@ufl.edu

DATE: 1/29/2020
TO: Manuela Corti
2004 Mowry Road - 2nd floor
Gainesville, Florida 32610
FROM: Peter Iafrate, IRB Chairman, University of Florida
Chair IRB-01
IRB#: **IRB201800502**
TITLE: Friedreich's Ataxia Gene Therapy Questionnaire

**Approved as Expedited**	**Expires on: 1/23/2023**

You have received IRB approval to conduct the above-listed research project. Approval of this project was granted on 1/23/2020 by IRB-01. This study is approved as expedited because it poses minimal risk and is approved under the following expedited category:

7. Research on individual or group characteristics or behavior (including, but not limited to, research on perception, cognition, motivation, identity, language, communication, cultural beliefs or practices and social behaviors) or research employing survey, interview, oral history, focus group, program evaluation, human factors evaluation or quality assurance methodologies. Note: Some research in this category may be exempt from the regulations for the protection of human subjects as noted in 45 CFR 46.101(b)(2) and (b)(3). This listing refers only to research that is not exempt.

Approval Includes, but is not limited to:

FA Questionnaire IRB Protocol

********Email Script**********

Consent Waiver Type:

Waiver of Documentation of Informed Consent

The researcher will still inform the potential subject about the research and seek to obtain consent, sometimes by including an IRB approved written statement that includes the mandatory elements of consent. However, consent of the subject is not documented by having the subject sign an Informed Consent form.

HIPAA Waiver Type:

to enroll subjects in the study

Principal Investigator Responsibilities:

The PI is responsible for the conduct of the study. Please review these responsibilities described at: http://irb.ufl.edu/irb01/researcher-information/researcherresponsibilities.html
Important responsibilities described at the above link include:

- Using currently approved consent form to enroll subjects (if applicable)
- Renewing your study before expiration
- Obtaining approval for revisions before implementation
- Reporting Adverse Events
- Retention of Research Records
- Obtaining approval to conduct research at the VA
- Notifying other parties about this project's approval status

Study Team:

Emma	Crowley	Study Coordinator
Samantha	Norman	Study Coordinator

The Foundation for The Gator Nation
An Equal Opportunity Institution
Confidentiality Notice: This e-mail message, including any attachments, is for the sole use of the intended recipient(s), and may contain legally privileged or confidential information. Any other distribution, copying, or disclosure is strictly prohibited. If you are not the intended recipient, please notify the sender and destroy this message immediately. Unauthorized access to confidential information is subject to federal and state laws and could result in personal liability, fines, and imprisonment. Thank you.
